# Combining Physiology-Based Modeling and Evolutionary Algorithms for Personalized, Noninvasive Cardiovascular Assessment Based on Electrocardiography and Ballistocardiography

**DOI:** 10.3389/fphys.2021.739035

**Published:** 2022-01-12

**Authors:** Nicholas Mattia Marazzi, Giovanna Guidoboni, Mohamed Zaid, Lorenzo Sala, Salman Ahmad, Laurel Despins, Mihail Popescu, Marjorie Skubic, James Keller

**Affiliations:** ^1^Department of Electrical Engineering and Computer Science, University of Missouri, Columbia, MO, United States; ^2^Department of Mathematics, University of Missouri, Columbia, MO, United States; ^3^Centre de Recherche Inria Saclay-Ile de France, Palaiseau, France; ^4^Department of Surgery, School of Medicine, University of Missouri, Columbia, MO, United States; ^5^Sinclair School of Nursing, University of Missouri, Columbia, MO, United States; ^6^Department of Health Management and Informatics, School of Medicine, University of Missouri, Columbia, MO, United States

**Keywords:** cardiovascular physiology, cardiovascular monitoring, ballistocardiography, physiology-based modeling, evolutionary algorithm (EA), personalized modeling, cuffless blood pressure estimation

## Abstract

**Purpose:** This study proposes a novel approach to obtain personalized estimates of cardiovascular parameters by combining (i) electrocardiography and ballistocardiography for noninvasive cardiovascular monitoring, (ii) a physiology-based mathematical model for predicting personalized cardiovascular variables, and (iii) an evolutionary algorithm (EA) for searching optimal model parameters.

**Methods:** Electrocardiogram (ECG), ballistocardiogram (BCG), and a total of six blood pressure measurements are recorded on three healthy subjects. The R peaks in the ECG are used to segment the BCG signal into single BCG curves for each heart beat. The time distance between R peaks is used as an input for a validated physiology-based mathematical model that predicts distributions of pressures and volumes in the cardiovascular system, along with the associated BCG curve. An EA is designed to search the generation of parameter values of the cardiovascular model that optimizes the match between model-predicted and experimentally-measured BCG curves. The physiological relevance of the optimal EA solution is evaluated a posteriori by comparing the model-predicted blood pressure with a cuff placed on the arm of the subjects to measure the blood pressure.

**Results:** The proposed approach successfully captures amplitudes and timings of the most prominent peak and valley in the BCG curve, also known as the J peak and K valley. The values of cardiovascular parameters pertaining to ventricular function can be estimated by the EA in a consistent manner when the search is performed over five different BCG curves corresponding to five different heart-beats of the same subject. Notably, the blood pressure predicted by the physiology-based model with the personalized parameter values provided by the EA search exhibits a very good agreement with the cuff-based blood pressure measurement.

**Conclusion:** The combination of EA with physiology-based modeling proved capable of providing personalized estimates of cardiovascular parameters and physiological variables of great interest, such as blood pressure. This novel approach opens the possibility for developing quantitative devices for noninvasive cardiovascular monitoring based on BCG sensing.

## 1. Introduction

Cardiovascular diseases (CVDs) are disorders of the heart and blood vessels, including heart failure, stroke and hypertension, and represent the first leading cause of death worldwide (World Health Organization, 2021). Early detection and intervention of CVDs can reduce the number of preventable hospital readmissions, thereby helping patients maintain a better quality of life while significantly reducing healthcare costs (Wasfy et al., 2014; Soucier et al., 2018).

Cardiovascular function and oxygen delivery to the tissues depends on adequate hemoglobin stores, oxygen uptake from the lungs and cardiac output (CO). This delivery system relies on a complex interplay between the pumping action of the heart and the biomechanical properties of the vasculature (Chang et al., 2002; Vincent and De Backer, 2013). Thus, effective cardiovascular monitoring should provide a quantitative assessment of both cardiac and vascular functions (Holcroft et al., 2006; Vincent and De Backer, 2013). Traditional monitoring techniques, such as electrocardiography, echocardiography and intravascular catheterization, focus primarily on the heart, providing information on its electrical, mechanical, and fluid-dynamical functions. A complementary approach is offered by ballistocardiography, whose signal, the ballistocardiogram (BCG), captures the repetitive motion of the center of mass of the human body resulting from the blood motion within the circulatory system (Starr and Noordergraaf, 1967). Interestingly, the BCG signal reflects the status of the cardiovascular system as a whole, rather than the heart alone, thereby making it an ideal complement to traditional monitoring techniques. In addition, the acquisition of BCG signals is not invasive and does not require body contact, thereby eliminating the risk of infections and making it a viable option for both hospital and in-home monitoring.

The original device for BCG measurement used by Starr and others was a lightweight bed suspended by long cables (Starr and Noordergraaf, 1967). The blood flow of a subject lying on the suspended bed resulted in the bed swinging; the capture of the swing was the BCG signal. A replica of Starr's suspended bed has been built within the MU Center for Eldercare and Rehabilitation Technology (CERT) directed by Prof. Skubic. The suspended bed is an impractical device for BCG measurement, especially compared to the electrocardiogram (ECG), which can be taken on virtually any platform using electric leads placed on the body in a standard configuration. Recently, a resurgence of BCG research has occurred, as new sensing devices (e.g., in the form of bed sensors, chair sensors, weighing scales) allow easier, noninvasive capture of the BCG signal. Several of these sensors are now available commercially (Alametsä et al., 2008; Chen et al., 2008; Shin et al., 2008; Young et al., 2008; Inan et al., 2009, 2014; Giovangrandi et al., 2011; Heise et al., 2011; Satu and Jukka, 2012; Zimlichman et al., 2012; Paalasmaa et al., 2014; Helfand et al., 2016; Katz et al., 2016; Huffaker et al., 2018).

Deciphering the cardiovascular mechanisms that determine the shape of the BCG waveform in a particular individual is the key to fully unlocking the potential of BCG-based monitoring for noninvasive cardiovascular assessment. For example, Etemadi et al. showed that the relative time delay between the ECG and BCG peaks is an indicator of myocardial contractility (Etemadi et al., 2011). Su et al. (2018) showed that the changes in the amplitude of the systolic BCG peaks measured *via* a hydraulic bed sensor correlated with the change in blood pressure occurring pre- and post-exercise. In this study, we investigate how to utilize the BCG waveform to estimate cardiovascular parameters specific to a given subject, which could be used for an in-depth assessment of cardiovascular function.

Specifically, we utilize the mathematical model proposed in Guidoboni et al. (2019) to simulate the BCG waveform based on fundamental principles of cardiovascular physiology. The model parameters quantify aspects of cardiovascular function that are particularly relevant for CVD monitoring, such as ventricular elastances and arterial stiffness, and the model simulations yield predictions of cardiovascular variables, such as blood pressure and volumes, and the resulting BCG waveform (Guidoboni, 2020). In Guidoboni et al. (2019), however, model parameters were chosen as representative of an idealized individual based on published literature. In this study, we investigate the use of an evolutionary algorithm (EA) to obtain *personalized estimates* of the cardiovascular model parameters based on the comparison between model-predicted and experimentally-measured BCG curves on a specific subject.

Ground truth for the cardiovascular parameters estimated *via* the EA is not easily available, and this constitutes the major challenge of this study. Some parameters may be estimated *via* noninvasive techniques (e.g., Doppler imaging can be used for arterial radii and lengths), which could be utilized on both healthy individuals and patients with CVD. Conversely, some parameters can only be estimated *via* highly invasive and risky procedures (e.g. ventricular catheterization is needed to assess end-systolic and end-diastolic elastances in the ventricles), which can only be performed when required by the specific health conditions of a patient with CVD. Thus, in order to properly design a study involving multiple techniques to measure cardiovascular parameters on healthy subjects and patients with CVD that could serve as ground truth to validate our EA findings, it is important to assess beforehand which of the many model parameters may be effectively estimated by the proposed EA method. This constitutes the specific goal of the present investigation.

The physiological relevance of the personalized solution is evaluated a posteriori by comparing the blood pressure estimated *via* the personalized model with the blood pressure measured via a cuff placed on the arm of the subject. When tested on three healthy individuals, the proposed EA performed better in estimating the parameters characterizing the function of the left ventricle than those of the right ventricle. The EA also performed well in estimating arterial stiffness. The satisfactory agreement between model-predicted and experimentally-measured values of blood pressure support the physiological relevance of these findings and show promise for utilizing the proposed approach as a quantitative method for noninvasive cardiovascular monitoring.

## 2. Methods

In this section 2, we outline the details on the signal acquisition (section 2.1), the physiology-based cardiovascular model (section 2.2), and the design of the EA algorithm (section 2.3).

### 2.1. Signal Acquisition and Processing for ECG, BCG, and Blood Pressure

Three healthy subjects were recruited for data collection in controlled laboratory settings. The sex, age, weight, and height of the subjects are summarized in Table 1. The subjects were asked to lie still on a suspended bed system as previously described in Guidoboni et al. (2019), while the ECG and BCG signals were recorded. The ECG was acquired *via* a 3-lead configuration and the BCG was acquired with a three-axis accelerometer from Kionix with 1,000 mV/g sensitivity placed on the bed frame of a suspended bed (Kionix, 2014). The ECG and BCG signals were collected simultaneously using an AD Instrument PowerLab 16/35 data acquisition system (ADInstruments, 2014). The ECG and BCG signals have been filtered *via* a 6th order Butterworth bandpass filter to remove the low-frequency respiratory motion and the high-frequency noise. Cut-off frequencies of 0.7–40 Hz and 1.25–15 Hz have been used for the ECG and BCG signals, respectively (Enayati, 2019).

**Table 1:** Summary of the participant information involved in this study.

**Subject**	**Sex**	**Age**	**Weight [kg]**	**Height [cm]**
1	Male	25	72.6	189
2	Male	32	72.6	180
3	Male	22	66.2	176

**Figure 1:**
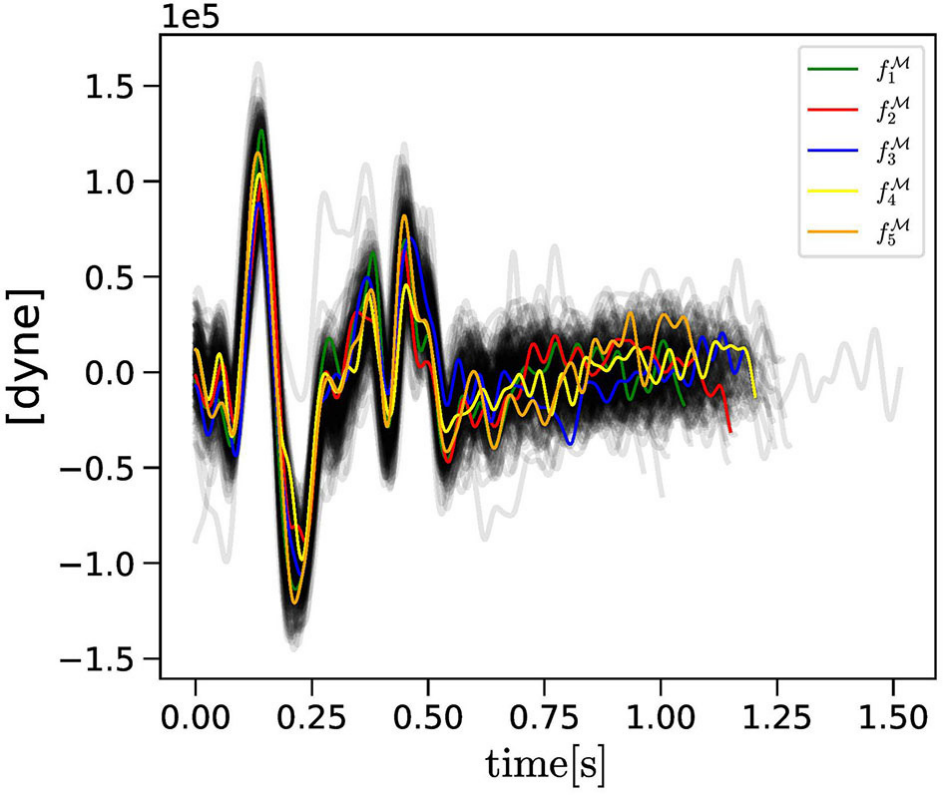
The collection $T_{f}^{M}$ of BCG curves measured experimentally (gray curves) are reported along with the $f_{k}^{M}$, with *k* = 1, …, *N*_*c*_ = 5, consecutive curves selected randomly as objective curves for the evolutionary algorithm (EA) applied to Subject 1.

The R peaks in the ECG, located *via* the Pan-Tompkins algorithm (Pan and Tompkins, 1985), were used to segment the BCG signal. Thus, a family of BCG curves is obtained, as shown in Figure 1. Let us denote by $T_{f}^{M}$ the family of all the BCG curves and by $f^{M}=f^{M}\left(t\right)$ a single BCG curve in the family, so that $f^{M}\in T_{f}^{M}$. The superscript M indicates that these curves are *measured*, as opposed to those that are predicted by the mathematical model (as shown in section 2.2). We note that $f^{M}$ has the units of a force [dyne] as it is obtained *via* the following relationship:


(1) $$\begin{array}{l} f^{M}\left(t\right)=m\times a\left(t\right)\;\text{[dyne]} \end{array}$$

where *m* is the mass of the subject (*m* = 74.2 Kg for the subject considered in this study) and *a*(*t*) is the acceleration [cm/s^2^] obtained by applying the following conversion to the signal *a*^*V*^(*t*) [V] actually measured by the accelerometer:


(2) $$\begin{array}{l} a\left(t\right)=G\frac{a^{V}\left(t\right)-\text{offset}}{S} \end{array}$$

with offset = 2.5 mV, *S* = 1 mV, and *G* = 981 cm/s^2^ (Kionix, 2014). Since the length of a cardiac cycle may vary from beat to beat, the length of each BCG curve may not be constant. Thus, the *k*−th curve $f_{k}^{M}\in T_{f}^{M}$ is defined for *t* ∈ [0, *T*_*c*_*k*__], where *T*_*c*_*k*__ is the length of the *k*−th cardiac cycle computed as the distance between two consecutive R peaks in the ECG.

In addition, the blood pressure was measured *via* a cuff placed on the arm of the subjects. A total of six blood pressure measurements were performed, three before the beginning of the ECG and BCG data acquisition and three afterward. An interval of 5 min was allowed between measurements. It is important to emphasize that cuff-based blood pressure measurements may interfere with the accelerometer-based BCG acquisition, as they introduce spurious movements. Furthermore, it is likely to expect that the blood pressure may decrease as the subject rests on the bed for a prolonged period of time. Thus, we adopted a protocol for blood pressure measurements to be performed both before and after the ECG-BCG data acquisition on the suspended bed. Ultimately, for each of the six measurements, systolic and diastolic blood pressures (SBP, DBP) are recorded and the pulse pressure (PP) is computed as the difference between the two, so that PP = SBP−DBP. The average PP value over the six measurements for the same individual is used as a comparison with the model prediction, as illustrated in section 3.3.

### 2.2. Physiology-Based Cardiovascular Model for Prediction of Blood Pressures, Blood Volumes, and BCG Waveform

The physiology-based model presented in Guidoboni et al. (2019) is utilized to simulate the blood flow through the cardiovascular system and to predict the resulting BCG waveform. In this study, we mention only the features of the model that are relevant for its combination with the EA illustrated in section 2.3, directing the interested reader to Guidoboni et al. (2019) for the full details.

In the model, the pumping action of the ventricles is described by the pressure generators


(3) $$\begin{array}{l} U_{L}\left(t\right)=\text{ULO }a_{L}\left(t\right),\;U_{R}\left(t\right)=\text{URO }a_{R}\left(t\right) \end{array}$$

where the subscripts *L* and *R* indicate the left and right ventricles, ULO and URO are positive constants representing the pressure build-up capacity in the ventricles, and *a*_*L*_(*t*) and *a*_*R*_(*t*) represent nondimensional activation functions for the timing of ventricular contractions defined as


(4) $$\begin{array}{l} a_{L}\left(t\right)=\frac{\tanh\left({\text{q}}_{L}t_{a}\right)-\tanh\left({\text{q}}_{L}t_{b}\right)}{2},\\ a_{R}\left(t\right)=\frac{\tanh\left({\text{q}}_{R}t_{a}\right)-\tanh\left({\text{q}}_{R}t_{b}\right)}{2} \end{array}$$

for *t*_*m*_ = mod(*t, T*_*c*_) < *T*_*s*_ and *a*_*L*_(*t*) = *a*_*R*_(*t*) = 0, otherwise. In Equation (4), *t*_*a*_ = *t* − *T*_*a*_ and *t*_*b*_ = *t* − *T*_*b*_, with *T*_*a*_, *T*_*b*_, q_*L*_, and q_*R*_ positive constants. Furthermore, *T*_*s*_ is the length of the systolic part of the cardiac cycle, and *T*_*c*_ is the length of the entire cardiac cycle. Interestingly, *T*_*c*_ can be tailored to a specific individual by means of the distance between R peaks in the ECG. The pressure generators defined in Equation (3) are connected in series with time-varying elastances defined as


(5) $$\begin{array}{l} E_{L}\left(t\right)=\text{ELD}+\text{ELS}a_{L}\left(t\right),\;E_{R}\left(t\right)=\text{ERD}+\text{ERS}a_{R}\left(t\right) \end{array}$$

where ELD, ELS, ERD, and ERS are positive constants.

Large arteries in the model play a very important role, as they are major contributors to the BCG waveform. Specifically, the model explicitly includes the ascending aorta (*i* = 2), the aortic arch (*i* = 3), the thoracic aorta (*i* = 4), the abdominal aorta (*i* = 5), the iliac arteries (*i* = 6), and the cerebral arteries (*i* = 14). The *i* labels follow those utilized in Guidoboni et al. (2019). In this study, for ease of reference, we define the set I={2,3,4,5,6,14} to indicate all the arteries in the model. Important parameters for each large artery *i*, for i∈I, are the radius *r*_*i*_ and the length *l*_*i*_. All arteries are assumed to have the same Young modulus *E* characterizing their stiffness. In the model, the iliac arteries in the systemic circulation are followed by the resistance *R*_7_ representing the peripheral vascular resistance. Finally, leveraging the electric analogy to fluid flow (Sacco et al., 2019), the model is completed by other capacitors, resistors, and inductors representing the microcirculation and the venous return to the heart.

The outputs of the physiology-based closed-loop model summarized above are the time-dependent distributions of pressures and volumes of blood as it flows in all vascular compartments. In particular, the computed blood volume in the left ventricle as a function of time allows us to calculate the end-diastolic volume (EDV), the end-systolic volume (ESV), the stroke volume (SV), the CO, and the ejection fraction (EF). Furthermore, the computed volume waveforms can be used to obtain the BCG waveform $f^{P}\left(t\right)$ as the acceleration of the center of mass of the human body resulting from the motion of blood through the cardiovascular system. The superscript P indicates that this waveform is model-predicted, as opposed to those measured experimentally (as shown in section 2.1). Specifically, $f^{P}\left(t\right)$ is computed as


(6) $$\begin{array}{l} f^{P}\left(t\right)=\rho_{b}\underset{n\in N}{\sum }\frac{d^{2}V_{n}}{dt^{2}}\left(t\right)y_{n}\;\text{[dyne]} \end{array}$$

where ρ_*b*_ is the blood density, the waveforms *V*_*n*_(*t*) represent the blood volume occupying the cardiovascular compartment *n* at time *t*, with n∈N=I∪{lv,rv}, and *y*_*n*_ represent the distance in the head-to-toe direction between the cardiovascular compartment *n*, with n∈N, and the plane of the heart valves. We note that N comprises the left and right ventricles, i.e., {*lv, rv*}, in addition to the large arteries, i.e., I. The values of all model parameters that are not explicitly estimated *via* the EA described in section 2.3 are assumed to be the same as those reported in Guidoboni et al. (2019).

### 2.3. Evolutionary Algorithm for Searching Personalized Parameters in the Physiology-Based Cardiovascular Model

An EA is utilized to search for the parameter values of the cardiovascular model described in section 2.2 that yield a satisfactory match between the model-predicted and experimentally-measured BCG waveforms, which we denoted by $f^{P}\left(t\right)$ and $f^{M}\left(t\right)$, respectively, for a given individual. We recall that an EA is a computational technique that abstracts from the mechanisms of evolution to search for optimal solutions to a problem. The EA search mechanism is inspired by Darwin's Theory of Evolution: similar to individuals evolving inside a species according to their fitness in the environment, solutions in an EA evolve in the search space in order to optimize an objective function (Ranganathan et al., 2018).

The *genotype* G in our EA comprises the set of parameters in the cardiovascular model whose optimal values are subject to search. In the following, we will denote by g∈G a specific choice for the parameter values, also referred to as *genetic coding*. Mutation rules will vary depending on the anatomical and physiological meaning of each parameter in G. To this end, it is convenient to write G as the union of the parameter sets pertaining to:

- The radii and lengths of the major arterial segments in the model, defined as $G^{r}=\left\{r_{i},i\in I\right\}$ and $G^{l}=\left\{l_{i},i\in I\right\}$,
- The coordinates of the cardiovascular compartments with respect to the heart valves, defined as $G^{y}=\left\{y_{n},n\in N\right\}$,
- The function of the left ventricle, defined as $G^{lv}=\left\{\text{ELS},\text{ELD},\text{ULO},{\text{q}}_{L},T_{s}\right\}$,
- The function of the right ventricle, defined as $G^{rv}=\left\{\text{ERS},\text{ERD},\text{URO},{\text{q}}_{R}\right\}$,
- The function of the vasculature, defined as $G^{vas}=\left\{E,R_{7}\right\}$.

Finally, we can write $G=G^{r}\cup G^{l}\cup G^{y}\cup G^{lv}\cup G^{rv}\cup G^{vas}$. A summary of the model parameters in the genotypes that are subject to the EA search is provided in Table 2.

**Table 2:** Summary of the model parameters in the genotypes considered in the evolutionary algorithm (EA).

**Genotype**	**Symbol**	**Unit**	**Description**
$G^{r}$	$r_{i},i\in I$	cm	arterial radii
$G^{l}$	$l_{i},i\in I$	cm	arterial lengths
$G^{y}$	$y_{n},n\in N$	cm	coordinates for BCG calculation
$G^{lv}$	ELS	mmHg cm^−3^	left ventricular end-systolic elastance
	ELD	mmHg cm^−3^	left ventricular end-diastolic elastance
	ULO	mmHg	pressure build-up capacity in the left ventricle
	q_*L*_	s^−1^	strength of left-ventricular activation
	*T*_*s*_	s	length of the systolic part of the cardiac cycle
$G^{rv}$	ERS	mmHg cm^−3^	right ventricular end-systolic elastance
	ERD	mmHg cm^−3^	right ventricular end-diastolic elastance
	URO	mmHg	pressure build-up capacity in the right ventricle
	q_*R*_	s^−1^	strength of right-ventricular activation
$G^{vas}$	*E*	mmHg	arterial young modulus
	*R*_7_	mmHg cm^−3^ s	peripheral resistance

The *phenotype* P represents the manifestation of a given genotype which, in our case, is given by the outputs of the cardiovascular model. In the following, we will denote by p∈P a specific phenotypic instance. The objective function directing the EA search focuses only on the fitness of the BCG waveform $f^{P}\left(t\right)$, but additional cardiac variables are used to ensure that the search results are physiologically acceptable. Specifically, we write $P=P^{fit}\cup P^{acc}$, with $P^{fit}=\left\{f^{P}\left(t\right),t\in \left[0,T_{c}\right]\right\}$ and $P^{acc}=\left\{\text{EDV},\text{ESV},\text{SV},\text{CO},\text{EF}\right\}$.

**Figure 2:**
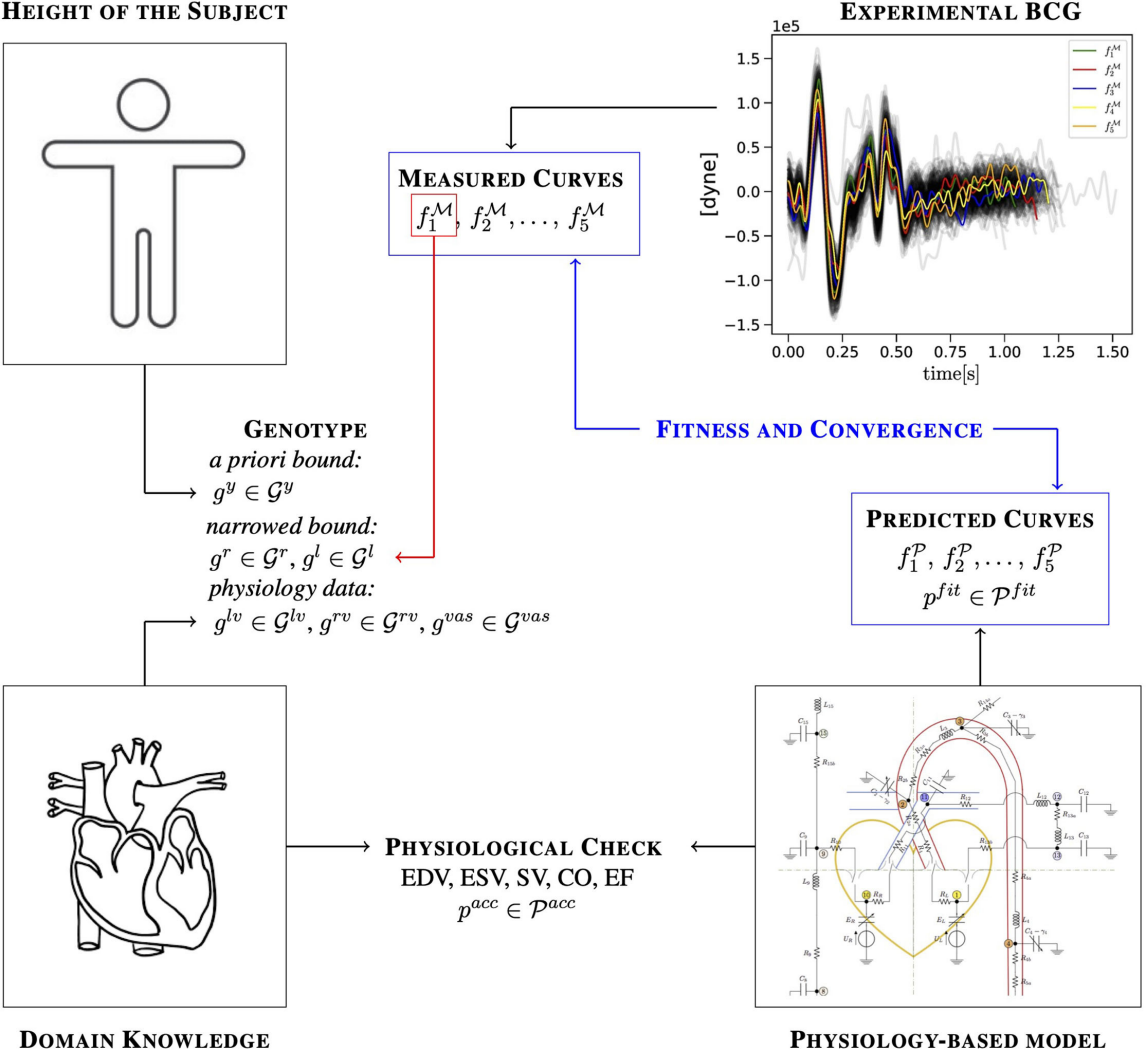
Illustration of how a priori knowledge about the subject, domain knowledge, experimental measurements, and predictions of a physiology-based mathematical model are combined in the EA to identify cardiovascular parameters for a specific subject.

The overall EA strategy is illustrated in Figure 2. We consider *N*_*c*_ consecutive curves $f_{k}^{M}\left(t\right)$, with *k* = 1, …, *N*_*c*_ and *t* ∈ [0, *T*_*c*_*k*__] as objective curves for the evolutionary search. An alternative choice could have been to create a single template representing the whole curve bundle. We opted for selecting *real* curves rather than a template in order to preserve the natural curve features as much as possible, including their different temporal lengths, while considering multiple curves in order to capture, rather than discard, beat-to-beat variations. In this study, we considered *N*_*c*_ = 5.

It is a physiological fact that no beat is exactly equal to next in any given person. As a consequence, it is physiological to expect that part of the genetic code G will present beat-to-beat variations and should, therefore, be treated independently when applying the EA algorithm to the *N*_*c*_ objective curves selected for the search. This is the case for $G^{lv}$, $G^{rv}$, and $G^{vas}$, which characterize the strengths of the ventricle contractions and the response of the vasculature. The mean radii, lengths, and locations of the major arteries, on the other hand, may vary with age or with the onset of disease conditions but are not expected to vary over the few minutes required for the BCG acquisition. To account for these differences in the expected genotype variations, we proceed as follows. We select one of the *N*_*c*_ objective curves and we apply the EA to search for $G^{r}$, $G^{l}$, and $G^{y}$ in addition to $G^{lv}$, $G^{rv}$, and $G^{vas}$. The values for $g^{r}\in G^{r}$ and $g^{l}\in G^{l}$ obtained during this preliminary search are then used to narrow down the interval for the radius and length variations considered when applying the EA to the remainder of the curves. In this study, we considered $f_{1}^{M}$ out of $f_{k}^{M}$, for *k* = 1, …, *N*_*c*_, for the preliminary search. The information about the height of the subject is used to limit the search range for $g^{y}\in G^{y}$ in all simulations. The strategy adopted in the EA implementation is detailed below. Whenever necessary, we will highlight the algorithm variations depending on whether it is applied to $f_{1}^{M}$ or to the remainder of the curves.

Step 1: Initial population. An initial population of *M* = 300 genotypes is obtained by computing mutations according to specific rules. In the following, we will denote by U(a,b) a uniform distribution in the range [*a, b*] and by N(0,σ) a normal distribution with mean equal to zero and SD equal to σ. Uniform distributions will be used in cases where upper and lower bounds for the range of parameter values can be set a priori, either by means of available measurements or domain knowledge. When such information is not available, a normal distribution is adopted. When the EA is applied to $f_{1}^{M}$, mutations are generated according to the following scheme:


(7) $$\begin{array}{l} g_{i}^{r}=U\left(g_{i,inf}^{r},\text{min}\left(g_{i,sup}^{r},g_{i-1}^{r}\right)\right)\;g_{i}^{r}\in G^{r}\;i=\left\{3,4,5,6\right\} \end{array}$$

(8) $$\begin{array}{l} g_{i}^{l}=U\left(g_{i,inf}^{l},g_{i,sup}^{l}\right)\;g_{i}^{l}\in G^{l}\;i\in I, \end{array}$$

(9) $$g_{n}^{y}=\{\begin{array}{l} U({\bar{g}}_{n}^{\;y},\alpha^{y}{\bar{g}}_{n}^{\;y})\text{ for }\alpha^{y}>1\\ U(\alpha^{y}{\bar{g}}_{n}^{\;y},{\bar{g}}_{n}^{\;y})\text{ for }\alpha^{y}<1 \end{array}\;g_{n}^{y}\in G^{y},\;n\in N.$$

(10) $$\begin{array}{l} g^{lv}={ḡ}^{lv}+N\left(0,\sigma^{lv}\right)\;g^{lv}\in G^{lv} \end{array}$$

(11) $$\begin{array}{l} g^{rv}={ḡ}^{rv}+N\left(0,\sigma^{rv}\right)\;g^{rv}\in G^{rv} \end{array}$$

(12) $$\begin{array}{l} g^{vas}={ḡ}^{vas}+N0,\sigma^{vas}g^{vas}\in G^{vas} \end{array}$$

Equations (7) and (8) show that radii and lengths for each of the major arteries, namely for each i∈I, are selected *via* a uniform distribution based on the ranges $\left[g_{i,inf}^{r},g_{i,sup}^{r}\right]$ and $\left[g_{i,inf}^{l},g_{i,sup}^{l}\right]$ reported in the literature (refer to Tables 4, 5). An additional anatomical constraint has been included in Equation (7) in order to ensure that that the radius decreases when moving down the arterial tree. Equations (10–12) show that the functional parameters for the ventricles and the vasculature are computed as mutations of the baseline values, indicated with bars, by means of normal distributions (as shown in Table 3). In this study, we assumed the SDs to be equal to half of the baseline values. Equation (16) shows that the genetic code $g^{y}\in G^{y}$ is selected *via* a uniform distribution between the baseline values ${ḡ}_{n}^{y}$, for i∈N and their scaled valued by means of a factor α^*y*^, which is assumed to be equal, larger, or less than 1 depending on whether the subject is as tall as, higher, or shorter than a reference height. In this study, we assumed the reference height to be 180 cm and we set α^*y*^ = 1.1 for Subject 1 (height: 189 cm), α^*y*^ = 1.0 for Subject 2 (height: 180 cm), and α^*y*^ = 0.85 for Subject 3 (height: 176 cm). In so doing, we leverage the available information on a specific subject to obtain an a priori estimate for $G^{y}$ and narrow its range for the EA search. All baseline values are the same as those reported in Guidoboni et al. (2019) except for *T*_*s*_, which has been estimated from the ECG recording as the average of the time interval between each R peak and the end of the following T wave.

**Table 3:** Summary of the baseline values and the SDs utilized in the EA simulation.

**Parameter**	**Unit**	**Baseline Value**	**Standard Deviation**
ELS	mmHg cm^−3^	1.375	0.6875
ELD	mmHg cm^−3^	0.04	0.02
ULO	mmHg	50	25
q_*L*_	s^−1^	6.28	3.14
*T*_*s*_	s	0.35	0.175
ERS	mmHg cm^−3^	0.23	0.115
ERD	mmHg cm^−3^	0.01	0.005
URO	mmHg	26	13
q_*R*_	s^−1^	6.28	3.14
E	mmHg	3000	1500
*R*_7_	mmHg cm^−3^ s	0.35	0.175

*All baseline values are the same as those reported in Guidoboni et al. (2019) except for T_s_, whose baseline value has been estimated from the ECG recording. The values for the SDs are assumed to be half of the baseline values*.

**Table 4:** Summary of the ranges for the arterial radii utilized in this study.

**Artery**	**Unit**	** ${\text{p}}_{i,inf}^{r}$ **	** ${\text{p}}_{i,sup}^{r}$ **	**Reference**
Ascending (*i* = 2)	[cm]	1.49	1.91	Wolak et al., 2008
Aortic arc (*i* = 3)	[cm]	1.14	1.42	Wolak et al., 2008
Thoracic (*i* = 4)	[cm]	0.92	1.28	Joh et al., 2013
Abdominal (*i* = 5)	[cm]	0.80	1.10	Joh et al., 2013
Iliac (*i* = 6)	[cm]	0.492	0.725	Joh et al., 2013
Carotid (*i* = 14)	[cm]	0.26	0.36	Krejza et al., 2006

**Table 5:** Summary of the ranges for the arterial lengths utilized in this study.

**Artery**	**Unit**	** ${\text{p}}_{i,inf}^{l}$ **	** ${\text{p}}_{i,sup}^{l}$ **	**Reference**
Ascending (*i* = 2)	[cm]	4	5	Goldman and Schafer, 2011
Aortic arc (*i* = 3)	[cm]	3.85	5.9	Boufi et al., 2017
Thoracic (*i* = 4)	[cm]	12.9	15.7	Redheuil et al., 2011
Abdominal (*i* = 5)	[cm]	13	16	Drake et al., 2009; Goldman and Schafer, 2011
Iliac (*i* = 6)	[cm]	3.7	7.5	Bergman, 2007
Carotid (*i* = 14)	[cm]	20	24.4	Choudhry et al., 2016

This procedure for determining the initial population is slightly modified when it is applied to each of the remaining selected curves $f_{k}^{M}$, for *k* = 2, …, *N*_*c*_. Specifically, *g*^*lv*^, *g*^*rv*^, *g*^*vas*^, and *g*^*y*^ are selected as in Equations (10–12, 16), whereas *g*^*r*^ and *g*^*l*^ are selected *via* a uniform distribution within a range of ±3% of the fittest genotypes obtained upon the convergence of the EA applied to $f_{1}^{M}$. Ultimately, this procedure gives the initial population of *M* genotypes, denoted as $g_{j}\in G$, with *j* = 1, …, *M*, that can be used to start the EA on each of the $f_{k}^{M}$ curves, with *k* = 1, …, *N*_*c*_, individually.

Step 2: Physiological check. For each of the genotypes *g*_*j*_ in the initial population, the corresponding phenotype *p*_*j*_, with *j* = 1, …, *M*, is computed *via* the physiology-based cardiovascular model. In order to be physiologically acceptable, we require the values of the cardiac variables in $P^{acc}$ to fall within some broad ranges reported in the literature and summarized in Table II of Guidoboni et al. (2019). The rationale behind the physiological check is that, in reality, the human body is capable of adapting ventricular and vascular parameters so that their combined action leads to proper cardiovascular function. Thus, not all randomly selected genotypes may lead to acceptable results. Even though this step may raise concerns about its applicability in disease conditions, the ranges utilized for the check are meant to be quite loose and only provide a way to exclude obviously erroneous genetic combinations. Thus, as more clinical and experimental data become available on the cardiac variables in $P^{acc}$ in health and disease, they can be used to enrich the domain knowledge and adjust the ranges for the physiological check. The generation of genotypes for the initial population continues till, after removal of unacceptable genotypes under the physiological check, a population of *M* = 300 physiologically-acceptable genetic codes is achieved.

Step 3: Fitness ranking. The phenotypes *p*_*j*_ are ranked according to their fitness, which we assume to be the similarity between the model-predicted BCG waveform $p_{j}^{fit}\in P^{fit}$ and the selected objective curve $f_{k}^{M}$, for *k* = 1, …, *N*_*c*_. We remind that this is done independently for each of the *N*_*c*_ objective curves. In this study, the similarity is quantified by means of the Euclidean distance between the phenotypic and objective curves, which is computed as follows. The measured waveform $f_{k}^{M}\left(t\right)$ is not available in analytic form, but rather as a sequence of values $f_{k,s}^{M}=f_{k}^{M}\left(t_{s}\right)$ at discrete time instants *t*_*s*_ ∈ [0, *T*_*c*_*k*__] as provided by the accelerometer. Since, by construction, all EA-generated curves are defined over the same time interval [0, *T*_*c*_*k*__] as the experimentally-measured objective function $f_{k}^{M}$, the values of the EA curves at the time instants *t*_*s*_ can be easily calculated and will be denoted by $f_{k,s}^{P}$. Then, the Euclidean distance between the functions $f_{k}^{M}\left(t\right)$ and $f_{k}^{P}\left(t\right)$ is computed as the Euclidean distance *d* between their discrete versions $\left(t_{s},f_{k,s}^{M}\right)$ and $\left(t_{s},f_{k,s}^{P}\right)$ as


(13) $$\begin{array}{l} d_{k}=\sqrt{\underset{s}{\sum }{\left(f_{k,s}^{M}-f_{k,s}^{P}\right)}^{2}}\;\text{for }k=1,\ldots ,N_{c}. \end{array}$$

The best 100 curves according to the fitness ranking are selected as parents for offspring generation.

Step 4: Offspring generation. The offspring genotypes are produced as follows:


(14) $$\begin{array}{l} g_{i}^{r}=g_{i,p}^{r}+U\left(\left(g_{i,inf}^{r}-g_{i,p}^{r}\right),\left(\text{min}\left(g_{i,inf}^{r},g_{i-1}^{r}\right)-g_{i,p}^{r}\right)\right)\\ g_{i}^{r}\in G^{r}i=\left\{3,4,5,6\right\}, \end{array}$$

(15) $$\begin{array}{l} g_{i}^{l}=g_{i,p}^{l}+U\left(\left(g_{i,inf}^{l}-g_{i,p}^{l}\right),\left(g_{i,sup}^{l}-g_{i,p}^{l}\right)\right)\\ \;g_{i}^{l}\in G^{l}i\in I, \end{array}$$

(16) $$\begin{array}{l} g_{n}^{y}=\{\begin{array}{c} U\left({ḡ}_{n}^{y},\alpha^{y}{ḡ}_{n}^{y}\right)\;\text{for}\alpha^{y}>1\\ U\left(\alpha^{y}{ḡ}_{n}^{y},{ḡ}_{n}^{y}\right)\;\text{for}\alpha^{y}<1 \end{array}\\ \;g_{n}^{y}\in G^{y},n\in N \end{array}$$

(17) $$\begin{array}{l} g^{lv}=g_{p}^{lv}+N\left(0,\sigma^{lv}\right)\\ \;g^{lv}\in G^{lv} \end{array}$$

(18) $$\begin{array}{l} g^{rv}=g_{p}^{rv}+N\left(0,\sigma^{rv}\right)\\ \;g^{rv}\in G^{rv} \end{array}$$

(19) $$\begin{array}{l} g^{vas}=g_{p}^{vas}+N\left(0,\sigma^{vas}\right)\\ \;g^{vas}\in G^{vas} \end{array}$$

where the subscript *p* indicates the genetic code of the parent. We note that the interval of variation for $g_{n}^{y}$, with n∈N, is assumed to be the same for parents and offsprings. All offsprings undergo the physiological check described in Step 2. A total of λ = 3 physiologically-acceptable offsprings are produced by each parent. Finally, the physiologically-acceptable offpsrings and their parents are ranked according to their similarity to the objective curve $f_{k}^{M}$ under consideration and the fittest λ = 100 genotypes are selected to form the generation advancing in the evolution. Utilizing this procedure, we ensure that (i) the population size is kept constant at *M* = 300 through the generations, and that (ii) only the fittest individuals advance from one generation to the next.

**Figure 3:**
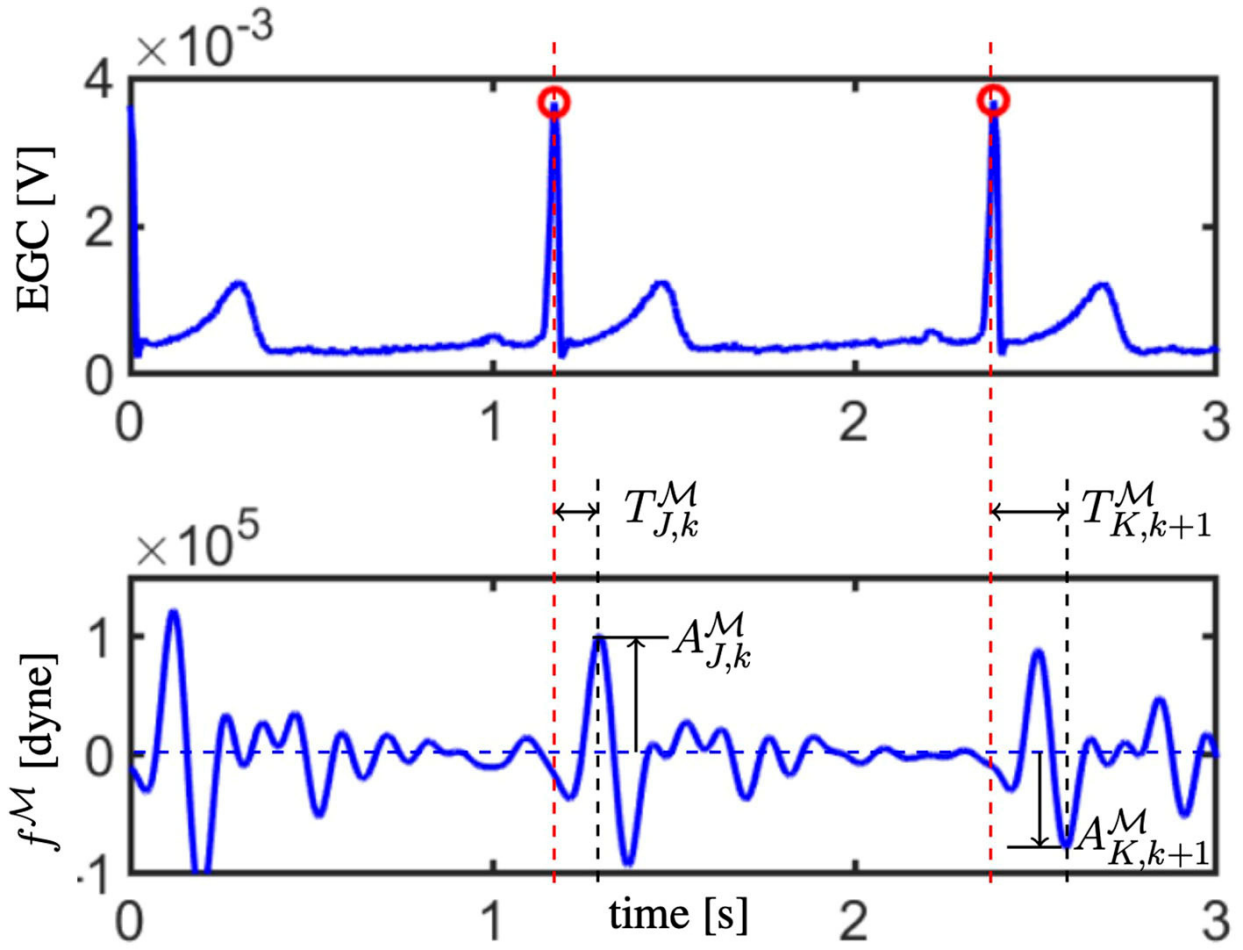
The electrocardiogam (ECG; top) and $f^{M}$ waveform (bottom) acquired synchronously are reported. R peaks in the ECG are marked with red circles and their time location is indicated by red dashed vertical lines. Amplitude and timing for the J peak are reported for the *k*−th curve (refer to $A_{J,k}^{M}$ and $T_{J,k}^{M}$), whereas the amplitude and timing for the K valley are reported for the following (*k* + 1)−th curve (refer to $A_{K,k+1}^{M}$ and $T_{K,k+1}^{M}$).

Step 5: Convergence check. The J peak and the K valley are among the most important traits characterizing the BCG waveform; they are detectable as the most prominent maximum and minimum following the R peak in the ECG (Starr and Noordergraaf, 1967). Let us denote by $A_{J,k}^{M}$ (resp. $A_{K,k}^{M}$) and $T_{J,k}^{M}$ (resp. $T_{K,k}^{M}$) the magnitude of the J peak (resp. K valley) and its timing with respect to the preceding R peak calculated for each of the *k* selected objective curves, with *k* = 1, …, *N*_*c*_. The location of the J peak and the K valley, along with their magnitudes and timings with respect to the R peaks in the ECG, are illustrated in Figure 3. The EA convergence is assessed by evaluating whether there is an offspring satisfying the following two criteria:

1. The predicted J-K magnitude and timings must be within a 5% range when compared to those computed for the objective curve under consideration;
2. The offspring must be within the top *N*_*fit*_ = 20 curves in the fitness ranking (as shown in Step 3).

We note that, by requiring the fitness ranking of the offspring to be high enough (second criterium), we aim at finding a solution that is close in an average sense to the measured curve while, simultaneously, maximizing the similarity with the J-K features (first criterium). If such an offspring exists, convergence is reached, otherwise, the EA continues to the next generation.

A maximum limit of 30 generations has been set as a stopping criterium in case convergence is not achieved.

Step 6: Solution features. Once the EA has reached convergence, the representative features of the EA solutions are computed as the average over the three best-ranked curves, namely


(20) $$\begin{array}{l} {\bar{A}}_{J,k}^{P}=\frac{1}{3}\overset{3}{\underset{b=1}{\sum }}A_{J,k}^{P,b}\;{\bar{A}}_{K,k}^{P}=\frac{1}{3}\overset{3}{\underset{b=1}{\sum }}A_{K,k}^{P,b} \end{array}$$

(21) $$\begin{array}{l} {\bar{T}}_{J,k}^{P}=\frac{1}{3}\overset{3}{\underset{b=1}{\sum }}T_{J,k}^{P,b}\;{\bar{T}}_{K.,k}^{P}=\frac{1}{3}\overset{3}{\underset{b=1}{\sum }}T_{K,k}^{P,b}. \end{array}$$

This holds for each EA run pertaining to the *k* = 1, …, *N*_*c*_ selected curves from the measured BCG.

Step 7: A posteriori blood pressure estimation. Ultimately, the EA provides a ***personalized estimate*** for the cardiovascular parameters summarized in Table 2 which, *via* the solution of the physiology-based model described in section 2.2, yields an estimate of the distributions of blood volumes and blood pressures within the cardiovascular system of a specific subject. To evaluate the reliability of these estimates, we compare the blood pressure values measured with a cuff directly on the subject, as described in section 2.1, with the blood pressure values predicted by the model with the personalized parameters. The cuff measures the pressure at the level of the brachial artery in the arm which, however, is not explicitly included among the major arteries of our cardiovascular model (as shown in section 2.2). To address this issue, we leverage the results of the Anglo-Cardiff Collaborative Trial, which included approximately 12, 000 individuals across East Anglia and Wales in the United Kingdom (McEniery et al., 2008). The study provides specific relationships that can be used to estimate the brachial pressure from the central aortic pressure. Interestingly, our cardiovascular models provide the central aortic pressure directly as the blood pressure in the ascending aorta (*i* = 2). In McEniery et al. (2008), differences in the diastolic values of the central aortic and brachial pressures were found to be negligible, thereby suggesting to assume the two values to be the same. Conversely, the systolic brachial values were found to be higher than the corresponding central aortic values, with specific increments and intervals of variability provided as a function of age and gender, as shown in Figure 1 of McEniery et al. (2008). Since in this study, we are considering three male subjects in the range of 20–29 years of age, we adopt an increment of 20 mmHg with an interval of variability of the ± 10 mmHg.

## 3. Results

We begin by comparing the BCG curves measured experimentally with those predicted by the EA algorithm (section 3.1). Next, we examine the EA performance in terms of estimating various parameters in the cardiovascular model (section 3.2). Finally, the values of PPs estimated by the algorithm are compared with those measured with the cuff placed on the arm of each subject (section 3.3).

### 3.1. Comparison Between Experimental and Predicted BCG Curves

**Figure 4:**
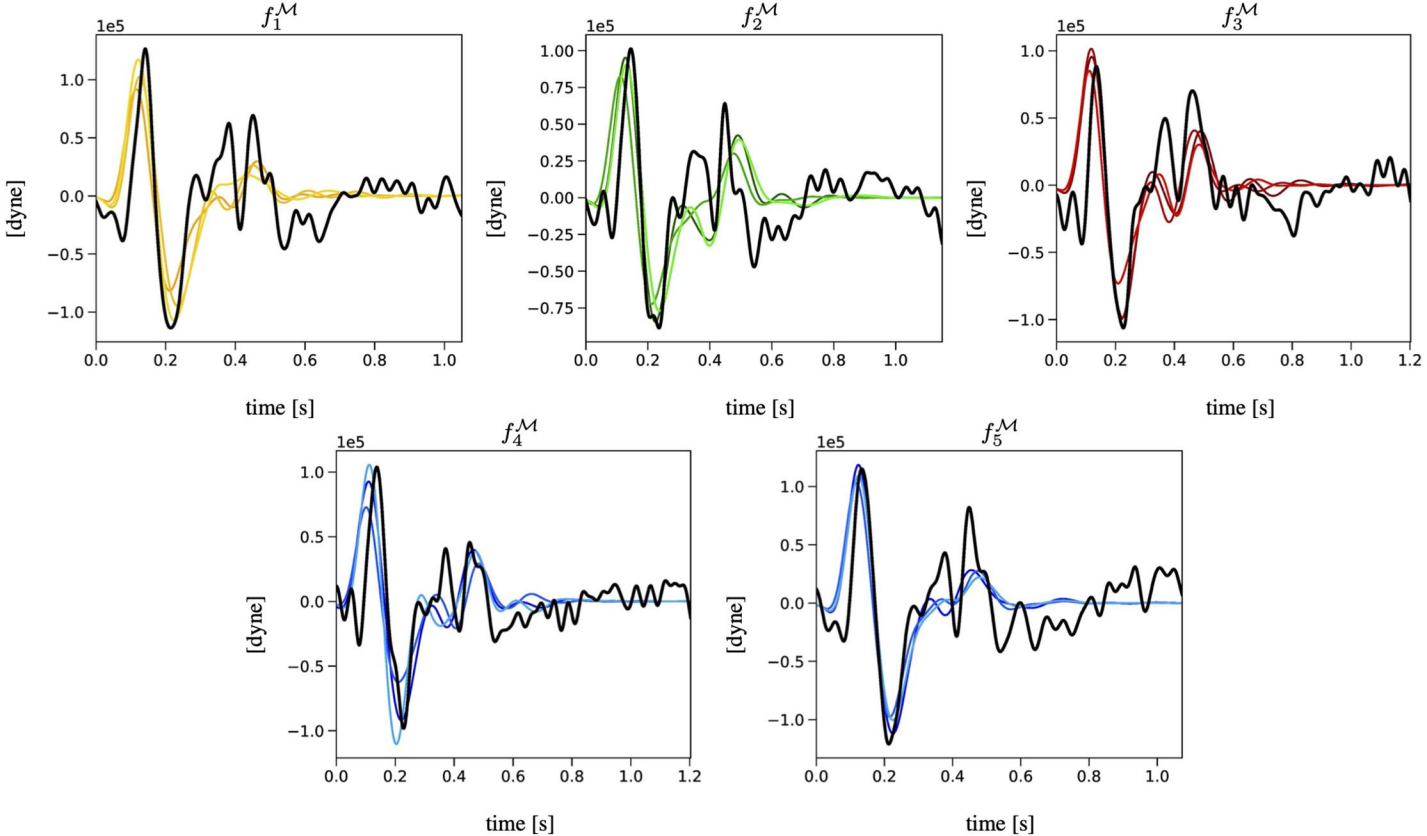
Comparison among the BCG curves $f_{k}^{M}$, with *k* = 1, …, 5 measured experimentally (in black) and the corresponding three best-ranked curves computed *via* the EA (in colors) for Subject 1.

Figure 4 reports the BCG curves $f_{k}^{M}$, with *k* = 1, …, 5 measured experimentally (in black) and the corresponding three best-ranked $f^{P}$ curves predicted *via* the EA (in colors) obtained for Subject 1. Analogous figures for Subjects 2 and 3 can be found in the Supplementary Material. Notably, the agreement between the measured and predicted curves in the systolic part of the cardiac cycle is quite satisfactory, with a clearly detectable similarity in terms of J-K features. During diastole, though, the predicted curves are much flatter than the measured curves, capturing only loosely the peaks and valleys that are exhibited experimentally. This result is not unexpected, since the diastolic features of BCG are known to be more challenging to capture both experimentally and theoretically (Starr and Noordergraaf, 1967; Guidoboni et al., 2019).

A quantitative comparison between the J-K features in the experimental and predicted curves for each of the three subjects included in the study is summarized in Table 6 by means of average percent errors in the amplitudes of the J and K peaks defined as


$$\begin{array}{l} \Delta \left({\bar{A}}_{J}^{P},A_{J}^{M}\right)=\frac{1}{5}\overset{5}{\underset{k=1}{\sum }}\frac{\left|{\bar{A}}_{J,k}^{P}-A_{J,k}^{M}\right|}{\left|A_{J,k}^{M}\right|}\times 100,\\ \Delta \left({\bar{A}}_{K}^{P},A_{K}^{M}\right)=\frac{1}{5}\overset{5}{\underset{k=1}{\sum }}\frac{\left|{\bar{A}}_{K,k}^{P}-A_{K,k}^{M}\right|}{\left|A_{K,k}^{M}\right|}\times 100 \end{array}$$

**Table 6:** Quantitative comparison between the J-K features in the experimentally-measured (M) and EA-predicted (P) curves.

**Features**	**Subject 1 (%)**	**Subject 2 (%)**	**Subject 3 (%)**
$\Delta \left({\bar{A}}_{J}^{P},A_{J}^{M}\right)$	12.9	15.2	21.1
$\Delta \left({\bar{A}}_{K}^{P},A_{K}^{M}\right)$	17.3	11.4	29.6
$\Delta \left({\bar{T}}_{J}^{P},T_{J}^{M}\right)$	1.7	1.2	1.5
$\Delta \left({\bar{T}}_{K}^{P},T_{K}^{M}\right)$	1.5	2.5	3.3

*Percent errors (Δ) in the amplitudes (A) and the timings (T) are computed for each subject*.

and in the timings of the J and K peaks defined as


$$\begin{array}{l} \Delta \left({\bar{T}}_{J}^{P},T_{J}^{M}\right)=\frac{1}{5}\overset{5}{\underset{k=1}{\sum }}\frac{\left|{\bar{T}}_{J,k}^{P}-T_{J,k}^{M}\right|}{\left|T_{J,k}^{M}\right|}\times 100,\\ \Delta \left({\bar{T}}_{K}^{P},T_{K}^{M}\right)=\frac{1}{5}\overset{5}{\underset{k=1}{\sum }}\frac{\left|{\bar{T}}_{K,k}^{P}-T_{K,k}^{M}\right|}{\left|T_{K,k}^{M}\right|}\times 100. \end{array}$$

Remarkably, the mean percent errors in the timings are approximately one order of magnitude lower than the percent errors in the amplitudes.

### 3.2. Estimation of Cardiovascular Parameters *via* the EA

**Figure 5:**
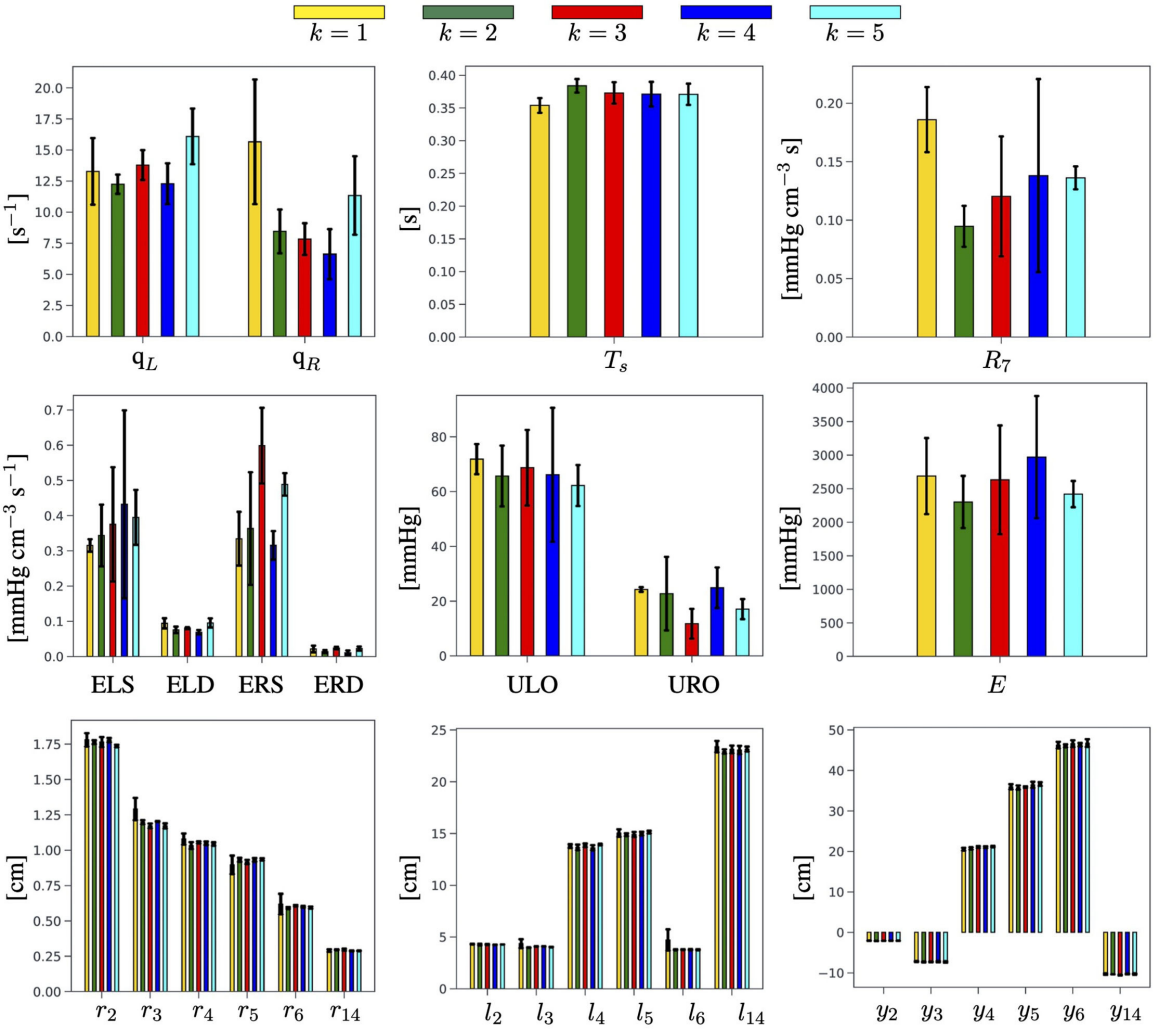
Summary of the physiological and anatomical parameters estimated by the EA for each of the *k* = 1, …, 5 selected BCG objective curves for Subject 1.

We recall that the main output of the EA algorithm is the personalized estimate of the physiological and anatomical parameters in G for a given subject. Detailed results are reported in Figure 5 in the case of Subject 1. Analogous figures for Subjects 2 and 3 can be found in the Supplementary Material. For each parameter, we consider the three best-ranked curves and we report the mean as the bar height, along with the maximum and the minimum values as black brackets. The results indicate that the proposed EA algorithm is capable of estimating in a consistent manner, over the five selected objective curves, all the parameters characterizing the left ventricle (ELS, ELD, ULO, q_*L*_, and *T*_*s*_) and the arterial Young modulus (*E*). Some of the parameters characterizing the right ventricle can also be estimated quite consistently (ERD, URO), while others show marked differences among the results obtained for the five curves (q_*R*_, ERS). Similar marked differences are displayed by the estimates for peripheral vascular resistance (*R*_7_). The values of some estimated parameters (*T*_*s*_, ULO, *E*, ERD, URO) turn out to be close to the baseline values reported in Table 3, while others deviate markedly. Interestingly, though, the EA estimates preserve relationships between relative parameter values without explicitly enforcing them, such as the facts that (i) ULO is larger than URO, implying that the capacity for pressure build-up in the left ventricle is larger than that in the right ventricle; and (ii) ELS (resp. ERS) is larger than ELD (resp. ERD), implying that the end-systolic elastance is larger than the end-diastolic elastance in both the left and right ventricles. Finally, the estimated values for radii, lengths, and locations of the arterial segments are also reported in Figure 3. They exhibit small differences as a result of the constraints imposed on the genotype generation.

A quantitative comparison of the cardiovascular parameters estimated *via* the proposed EA method for the three subjects included in the study is summarized in Table 7. For each estimated parameter, we report the mean value calculated over five objective curves, along with the minimum and maximum values (annotated in italics in parenthesis) obtained over all the curves for the same subject. Since a ground truth for the estimated parameters is not available, we utilized the width of the interval of the estimated parameters as an indicator of the EA potential for parameter estimation. More precisely, we utilized bold fonts to indicate in Table 7 those results for which the semidistance between the maximum and minimum is less than 1/3 of the estimated mean value. Interestingly, the estimates of ELD, ULO, *T*_*s*_, and URO satisfy this criterion for all three subjects, whereas the estimates of ELS, q_*L*_, ERS, q_*R*_, and E were satisfactory only for two out of three subjects. The value of the peripheral resistance *R*_7_ resulted to be poorly estimated in all subjects. It is also worth noticing that the results for all three subjects confirm that the ULO is estimated to be larger than URO, and that ELS (resp. ERS) is estimated to be larger than ELD (resp. ERD), thereby supporting the physiological relevance of the findings.

**Table 7:** Summary of EA estimated parameters for the three subjects involved in the study.

**Parameter**	**Unit**	**Subject 1**	**Subject 2**	**Subject 3**
ELS	mmHg cm^−3^	**0.37** ***(0.32,0.43)***	**0.37** ***(0.28,0.48)***	0.78 *(0.50,1.50)*
ELD	mmHg cm^−3^	**0.08** ***(0.07,0.10)***	**0.09** ***(0.08,0.10)***	**0.06** ***(0.04,0.08)***
ULO	mmHg	**66.92** ***(62.22,71.84)***	**80.22** ***(74.21,87.31)***	**74.23** ***(58.11,90.89)***
q_*L*_	s^−1^	**13.54** ***(12.25,16.10)***	**12.85** ***(10.48,16.02)***	15.76 *(3.51,26.08)*
*T*_*s*_	s	**0.37** ***(0.35,0.38)***	**0.37** ***(0.36,0.38)***	**0.37** ***(0.36,0.39)***
ERS	mmHg cm^−3^	0.42 *(0.32,0.60)*	**0.37** ***(0.30,0.52)***	**0.37** ***(0.26,0.42)***
ERD	mmHg cm^−3^	0.019 *(0.011,0.024)*	**0.018** ***(0.012,0.023)***	0.020 *(0.010,0.037)*
URO	mmHg	**20.15** ***(11.76,24.90)***	**21.73** ***(16.58,26.16)***	**23.95** ***(18.28,30.67)***
q_*R*_	s^−1^	9.98 *(6.62,15.66)*	**8.36** ***(6.48,10.42)***	**9.71** ***(7.84,11.34)***
E	10^3^ mmHg	**2.60** ***(2.30,2.97)***	**4.04** ***(3.45,4.88)***	4.60 *(3.33,8.22)*
*R*_7_	mmHg cm^−3^ s	0.14 *(0.09,0.19)*	0.19 *(0.14,0.27)*	0.11 *(0.06,0.16)*

*Mean values are reported along with the minimum and maximum values (in italics, in parenthesis). Bold fonts are used to indicate the instances for which the semidistance between maximum and minimum is less than 1/3 than the estimated mean value*.

### 3.3. Central Aortic Pressure and Brachial Pressure

**Figure 6:**
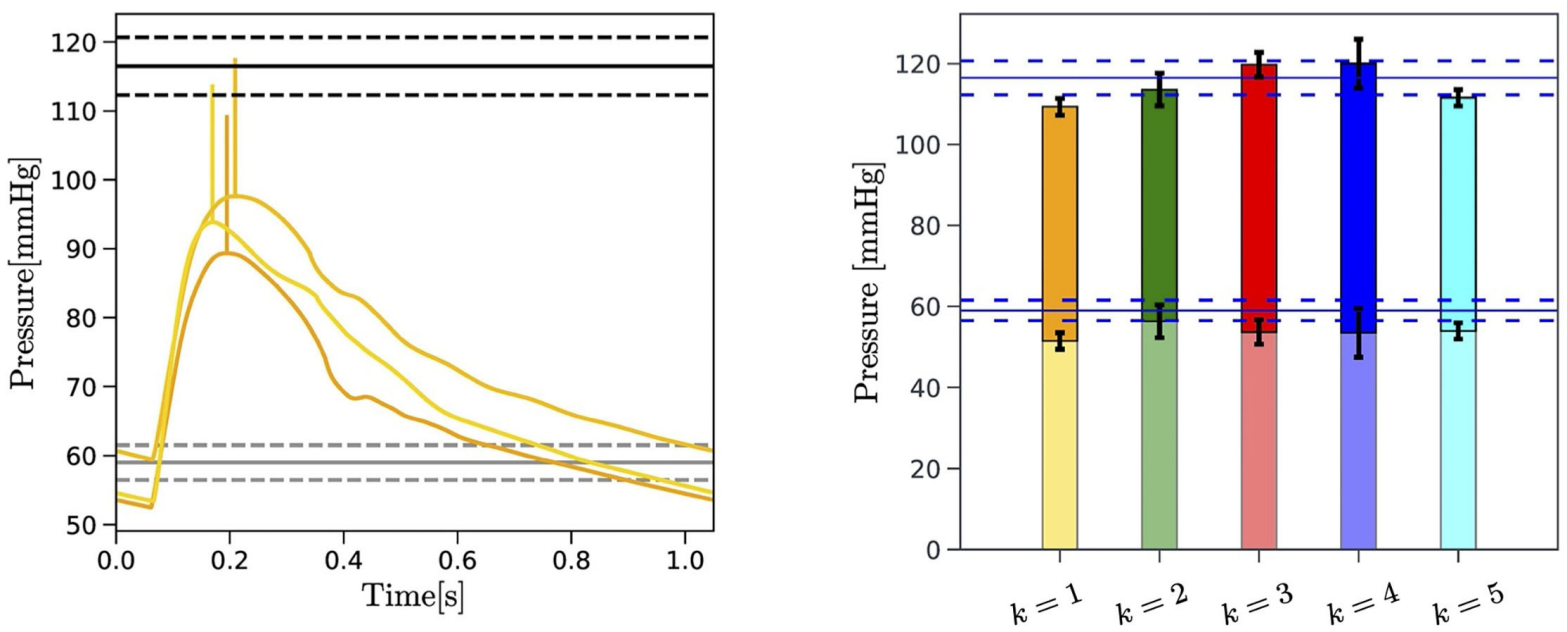
Left: Comparison between the central aortic pressure corresponding to the three best-ranked curves selected by the EA search performed on $f_{1}^{M}$ for Subject 1 (yellow curves) and the blood pressure measured at the arm with a cuff of the subject (horizontal black lines). The 20 mmHg increment applied to the systolic value of predicted central aortic pressure is also indicated (vertical yellow segments). The mean (solid black lines) maximum and minimum value (dashed black lines) of the repeated blood pressure measurements are reported. *Right:* Comparison between the experimentally measured brachial pulse pressure (PP) (horizontal blue lines) and the brachial pressure predicted by the EA for each of the *k* = 1, …, 5 objective curves for Subject 1. The PP is indicated with solid colors. Maximum and minimum values obtained for the three best-ranked curves for each $f_{k}^{M}$, with *k* = 1, …, *N*_*c*_, are reported in black brackets.

To further verify the capability of the EA algorithm to yield physiologically-meaningful solutions, we compare the brachial pressure measured experimentally with the pressure predicted by the cardiovascular model equipped with the personalized parameters provided by the EA search. The results obtained for Subject 1 are reported in Figure 6, where the horizontal lines indicate the mean (solid line), maximum, and minimum values (dashed lines) of the six systolic and diastolic pressure measurements acquired with a cuff placed on the arm of the subject (refer to section 2.1). Analogous figures for Subjects 2 and 3 can be found in the Supplementary Material. Figure 6 (*Left*) shows the pressure waveforms in the ascending aorta (*i* = 2) predicted by the cardiovascular model for the three best-ranked curves obtained by the EA performed on $f_{1}^{M}$. Following McEniery et al. (2008), we apply a 20 mmHg increment (vertical yellow segments) to the predicted systolic value of the central aortic pressure to estimate the systolic value of the brachial pressure. Differences in the diastolic values of the central aortic and brachial pressure are neglected. Figure 6 (*Right*) reports the results for the brachial pressure predicted by the cardiovascular model for Subject 1 with the personalized model parameters yielded by the EA search performed on each of the *k* = 1, …, 5 objective curves $f_{k}^{M}$. The height of the colored bars represents the mean value over the three best-ranked curves obtained for a given *k*, whereas the black brackets indicate the maximum and minimum values. The pulse pressure, defined as the difference between systolic and diastolic values, is highlighted with solid colors in the bars.

A comparison between the values of PP measured experimentally and predicted by the proposed EA method for each subject of three subjects is summarized in Table 8. The measured values correspond to the average PP values over a total of six measurements obtained with the cuff, as described in section 2.1. The values reported in italics in parenthesis indicate the minimum and maximum values in the single measurements. The EA-predicted values are obtained through the following steps: (i) a central aortic pressure waveform (refer to Figure 6) is obtained *via* the cardiovascular model (refer to section 2.2) with the set of model parameters corresponding to the three best-ranked curves; (ii) a factor of 20±10 mmHg is added to the systolic value of the simulated central aortic pressure to obtain the systolic brachial pressure, as suggested by the population-based study of McEniery et al. (2008) (refer to section 2.1); (iii) the PP is calculated as the difference between the estimated systolic brachial pressure and the diastolic blood pressure; (iv) the PP values are averaged over the three best-ranked curves for each of the five objective curves for each subject, with the overall minimum and maximum values reported in italics in parenthesis. Since a ground truth for the central aortic pressure is not available for this study, the fact that the predicted PP values are within the measured intervals for all three subjects is very promising and provides supportive indirect evidence that the parameters estimated *via* the proposed EA method actually bear physiological relevance.

**Table 8:** Comparison between the pulse pressure (PP) values measured *via* a cuff placed in the arm and the PP values predicted as a result of the cardiovascular parameters estimated *via* the proposed EA method.

**Pulse Pressure [mmHg]**	**Subject 1**	**Subject 2**	**Subject 3**
Measured	57.5	40.5	57.3
	*(53,62)*	*(35,47)*	*(52,60)*
Predicted	61.1	41.6	44.7
	*(51.1,71.1)*	*(31.6,51.6)*	*(34.7,54.7)*

*Variability intervals are reported in italics in parenthesis*.

## 4. Discussions

The novelty of the approach proposed in the present study consists in leveraging a physiology-based mathematical model to incorporate substantial domain knowledge in an EA whose objective is to attain optimal fitness between model-predicted and experimentally-measured BCG curves on a given subject (refer to Figure 4 and Table 6). By doing so, we are able to obtain personalized estimates of cardiovascular parameters (refer to Figure 5 and Table 7) and of variables of physiological interest, such as the central aortic and brachial pressures (see Figure 6 and Table 8).

In the current implementation of the algorithm, we opted for selecting *N*_*c*_ = 5 consecutive experimental BCG curves rather than a single template representing the whole data acquisition. This choice is motivated by the fact that amplitude, timing, and length of each BCG curve embody the action and function of the ventricles and the vasculature during a single heart-beat and may as well vary in the next. Thus, we do not expect the EA estimates for the personalized cardiovascular parameters to be the same from beat-to-beat, even for healthy subjects such as those considered in this study, but rather to be in the same ballpark, as shown in Figure 5. A limitation of the current study is that the values of the estimated parameters are not directly comparable to independent measurements. Radii, length, and locations of the main arteries, $G^{r}$, $G^{l}$, and $G^{y}$ could be acquired, for example, using Doppler imaging. Such information could either be used as a posterior verification of the EA predictions or as a priori knowledge that would narrow the EA search range for that specific subject. The measurement of other parameters, such as the ventricular elastances, requires invasive techniques based on catheterization. The capability of the proposed approach to yield physiologically-meaningful solutions is confirmed by the good agreement between the PP values predicted by the cardiovascular model personalized *via* the EA method and the experimental measurements (refer to Figure 6 and Table 8). This result is particularly encouraging, considering that the blood pressure values were *not* included in any of the feature sets utilized in the EA for the physiological check (refer to Step 2, section 2.3), fitness evaluation (refer to Step 3, section 2.3), and convergence (refer to Step 5, section 2.3). This finding suggests that our approach could be used to obtain BCG-based cuffless blood pressure measurements, along with noninvasive estimates of central aortic pressure and valuable parameters describing cardiovascular function.

In the long term, this study aims at contributing to the quest for noninvasive techniques capable of providing meaningful insights into the thermodynamic efficiency of cardiac function. Without direct left ventricular inductance catheters, clinicians must rely on indirect estimations from right heart catheters or algorithms from echocardiograms, each affected by risks and limitations (Ikonomidis et al., 2019). By providing noninvasive estimates of left-ventricular end-systolic elastance and central aortic pressure based on a mechanistic interpretation of the BCG signal, this study could provide clinicians with a rapid and insightful assessment of cardiac function that could be used at the bedside of the critically ill patient and offer practical solutions for outpatient monitoring. To get a sense of how these results could be used in practice, let us look at the parameters estimated for Subjects 1 and 2 in Table 7. Based on the EA-guided interpretation of the BCG signal, the pressure build-up capacity in the left ventricle (ULO) for Subject 2 is approximately 20% higher than in Subject 1, while being very close for the right ventricle (URO). This difference does not constitute a problem per se; rather, it shows how the BCG could be used to establish a cardiovascular baseline for each individual. In the case of outpatient monitoring, longitudinal measurements over the course of months could help detect a deterioration in left-ventricular function by, for example, providing a quantitative trajectory of decreasing ULO values. In the case of critically ill patients, frequent monitoring (possibly continuous) may be advisable in order to enable early detection of cardiogenic shock. Similarly, this method could be used to track changes in left-ventricular end-systolic and end-diastolic elastances (ELS, ELD), whose changes are indicative of heart failure with reduced and preserved EF, respectively (Guidoboni et al., 2019). Our group is currently conducting studies on human subjects and on swine to provide further data supporting the theoretical findings reported in the article, hopefully, bring us closer to making this vision a reality.

Limitations from both the experimental and modeling viewpoints should be considered when evaluating the findings of our study. The BCG sensing modality utilized in this study is a suspended bed equipped with an accelerometer. While providing a signal that is very close to the true acceleration of the center of mass of the human body (Starr and Noordergraaf, 1967), this sensing modality is primarily used in research laboratories and is not amenable to clinical or in-home use. Despite differing in shape among sensing modalities, all BCG waveforms exhibit a major peak (i.e., J peak) and a major valley (i.e., K valley) (Giovangrandi et al., 2011). Thus, by using only the J-K amplitudes and timings in the convergence of the EA algorithm, the approach described in this study could be extended to other BCG technologies. An ongoing study in the Surgical Intensive Care Unit (MU Health Care, Columbia, MO) has recently shown that measures of timing between ECG and BCG signals acquired on critically ill patients by means of a three-axis accelerometer positioned under the head pillow are feasible and reproducible (Zaid et al., 2021), thereby showing good potential for applications of the proposed methodology beyond a laboratory setting.

Additionally, our results show that the agreement between model-predicted and experimentally measured BCG curves is better in the systolic part than in the diastolic part of the cardiac cycle (refer to Figure 4). It is known that the BCG signal is stronger during systole when the ventricular contractions occur and the blood from the left ventricle is ejected and channeled through the aorta (Starr and Noordergraaf, 1967; Kim et al., 2016). Thus, the experimental measurements are much more reliable during systole than diastole. Furthermore, the physiological-based cardiovascular model for BCG prediction used in this study is capable of simulating the systolic peak and valleys of the BCG waveform with much greater accuracy than those in the diastole (Guidoboni et al., 2019). Due to these experimental and theoretical limitations, we based our convergence criteria on systolic features of the BCG waveform. In future studies, with the advances of BCG technologies and physiological understanding of the BCG waveform, these features could be extended to include also the diastolic part of the cardiac cycle. An aspect that could be considered in evaluating the performance of the proposed EA method is a different choice for *N*_*c*_ representing the number of consecutive BCG curves to be selected as objective curves. The choice of *N*_*c*_ = 5 adopted in this study is motivated by the need of considering multiple curves while maintaining the overall computational load affordable. Optimal choices for *N*_*c*_ may be explored in conjunction with the effect of breathing, which may affect the BCG curves over longer intervals.

## 5. Conclusions

This study presented a novel combination of a physiology-based mathematical model and an evolutionary algorithm to obtain personalized estimates of cardiovascular parameters and variables of physiological interest, such as blood pressure, with the goal of developing quantitative tools for noninvasive cardiovascular evaluations based on BCG sensing. The approach proved capable of estimating many ventricular and arterial parameters with consistency when five consecutive BCG curves were selected for the subjects considered in this study. Furthermore, the good agreement between the blood pressure estimated with the model and measured experimentally with a cuff shows that the proposed approach is physiologically meaningful and may provide theoretical support to the further development of cuffless methods for blood pressure measurements (Solà and Delgado-Gonzalo, 2019; Le et al., 2020; Pandit et al., 2020). Investigations evaluating the applicability of the proposed approach to situations where the data acquisition is not as controlled as in a laboratory setting are currently under-way. Preliminary results obtained when monitoring critically ill patients hospitalized in the Surgical Intensive Care Unit (University Hospital, MU Health Care System) suggest that measurements of BCG amplitudes and timings are feasible and reproducible (Zaid et al., 2021), thereby yielding promise for future extension of this study.

## Data Availability Statement

The original contributions presented in the study are included in the article/Supplementary Material, further inquiries can be directed to the corresponding author.

## Ethics Statement

The studies involving human participants were reviewed and approved by Institutional Review Board, Office of Research and Economic Development, University of Missouri. The patients/participants provided their written informed consent to participate in this study.

## Author Contributions

NM, LS, and MZ contributed to implementing the algorithm and running the numerical simulations. GG, NM, and MZ wrote the first draft of the manuscript. All authors contributed to the conception and design of the study, contributed to manuscript revision, read, and approved the submitted version.

## Conflict of Interest

GG would like to disclose that she received remuneration from Foresite Healthcare LLC for serving as a consultant. MS also discloses a conflict with Foresite Healthcare LLC outside the submitted work and patents licensed to Foresite Healthcare LLC. These relationships are pursuant to the University of Missouri's policy on outside activities. The remaining authors declare that the research was conducted in the absence of any commercial or financial relationships that could be construed as a potential conflict of interest.

## Publisher's Note

All claims expressed in this article are solely those of the authors and do not necessarily represent those of their affiliated organizations, or those of the publisher, the editors and the reviewers. Any product that may be evaluated in this article, or claim that may be made by its manufacturer, is not guaranteed or endorsed by the publisher.
