# Supplementary material for: Combining Physiology-Based Modeling and Evolutionary Algorithms for Personalized, Noninvasive Cardiovascular Assessment Based on Electrocardiography and Ballistocardiography
Source: Front Physiol. 2022 Jan 12;12:739035. doi: 10.3389/fphys.2021.739035 (PMC8790319; doi:10.3389/fphys.2021.739035)
Supplement: Supplementary file 1 [file Data_Sheet_1.PDF]

Supplementary Material

Table1 {Summary of EA estimated geometric parameters for the three subjects involved in the study. Mean values are reported along with the minimum and maximum values (in italics, in parenthesis)}

PARAMETER	UNIT	SUBJECT 1	SUBJECT 2	SUBJECT 3
**RASC**	[cm]	1.76 (1.74, 1.78)	1.78 (1.67 - 1.84)	1.66 (1.58 - 1.79)
**RARC**	[cm]	1.21 (1.17, 1.29)	1.31 (1.26 - 1.39)	1.32 (1.25 - 1.39)
**RTHOR**	[cm]	1.05 (1.03 - 1.08)	1.10 (0.98 - 1.22)	1.06 (1.00 - 1.15)
**RABD**	[cm]	0.92 (0.90 - 0.93)	0.91 (0.85 - 0.97)	0.95 (0.94 - 0.97)
**RILIAC**	[cm]	0.60 (0.59 - 0.62)	0.62 (0.55 - 0.67)	0.64 (0.58 - 0.67)
**RCAR**	[cm]	0.29 (0.29 - 0.30)	0.32 (0.30 - 0.33)	0.30 (0.27 - 0.33)
**LASC**	[cm]	4.27 (4.25 - 4.31)	4.45 (4.27 - 4.66)	4.60 (4.50 - 4.75)
**LARC**	[cm]	4.10 (3.98 - 4.36)	5.08 (4.61 - 5.46)	5.17 (5.01 - 5.30)
**LTHOR**	[cm]	13.77 (13.63 - 13.93)	13.99 (13.12 - 14.68)	14.17 (13.48 - 14.43)
**LABD**	[cm]	14.99 (14.89 - 15.14)	14.09 (13.29 - 15.01)	14.15 (13.79 - 14.38)
**LILIAC**	[cm]	3.96 (3.76 - 4.72)	5.39 (4.95 - 5.90)	5.55 (4.79 - 6.61)
**LCAR**	[cm]	23.13 (22.92 - 23.39)	22.10 (21.28 - 23.51)	21.98 (20.85 - 22.90)
**YASC**	[cm]	-2.07 (-2.09 - -2.04)	-1.78 (-1.9 - -1.7)	-2.10 (-2.19 - -2.03)
**YARC**	[cm]	-7.26 (-7.30 - -7.19)	-6.23 (-6.65 - -5.95)	-7.36 (-7.65 - -7.12)
**YTHOR**	[cm]	20.93 (20.55 - 21.19)	17.8 (17 – 19)	21.02 (20.34 - 21.86)
**YABD**	[cm]	36.15 (35.77 - 36.66)	31.15 (29.75 - 33.25)	36.79 (35.60 - 38.26)
**YILIAC**	[cm]	46.41 (46.07 - 46.76)	40.05 (38.25 - 42.75)	47.30 (45.77 - 49.19)
**YCAR**	[cm]	-10.34 (-10.53 - -10.25	-8.9 (-9.5 - -8.5)	-10.51 (-10.93 - -10.17)

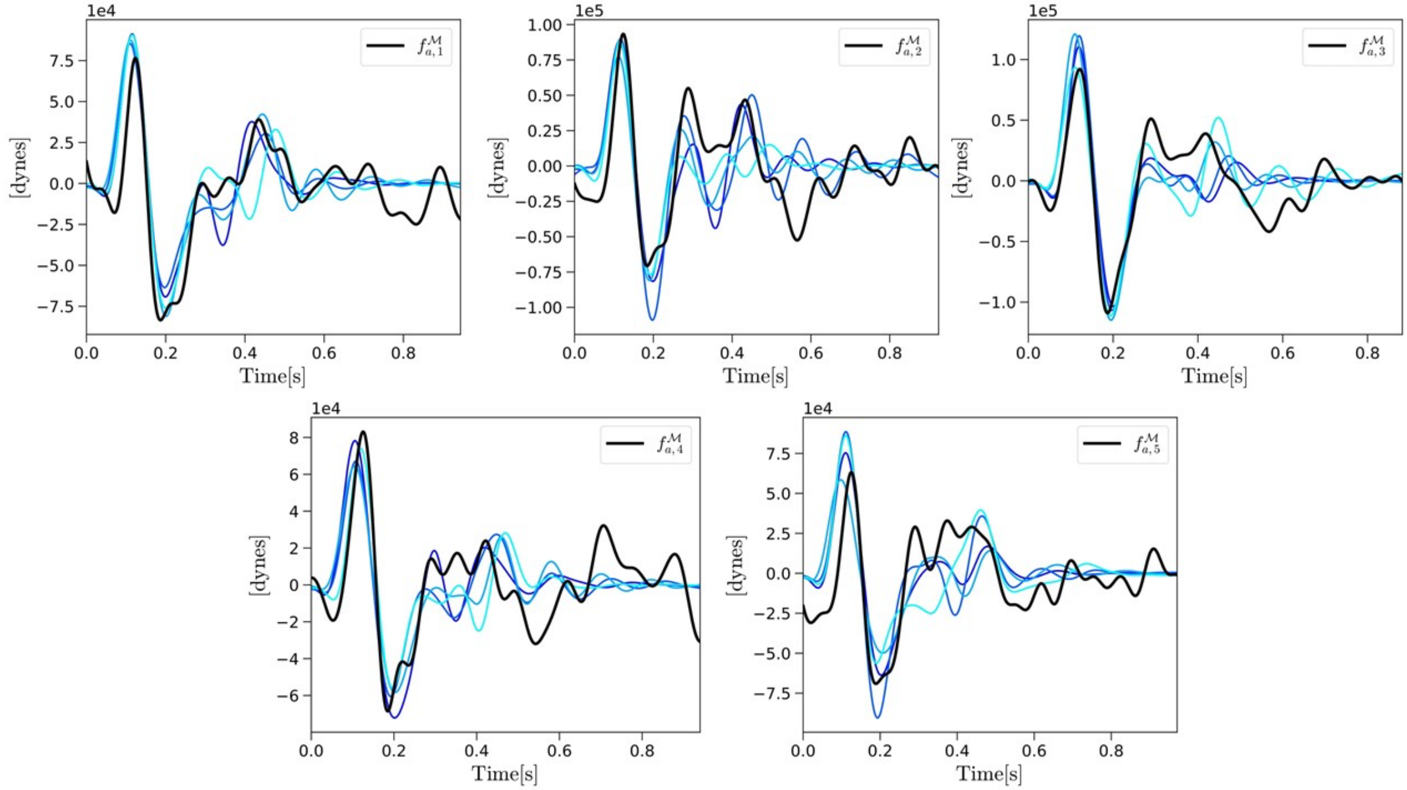

Figure 1 Comparison among the BCG curves $f^{M}{}_{k}$, withk= 1,...,5measured experimentally (in black)and the corresponding three best-ranked curves computed via the EA (in colors) for Subject 2.

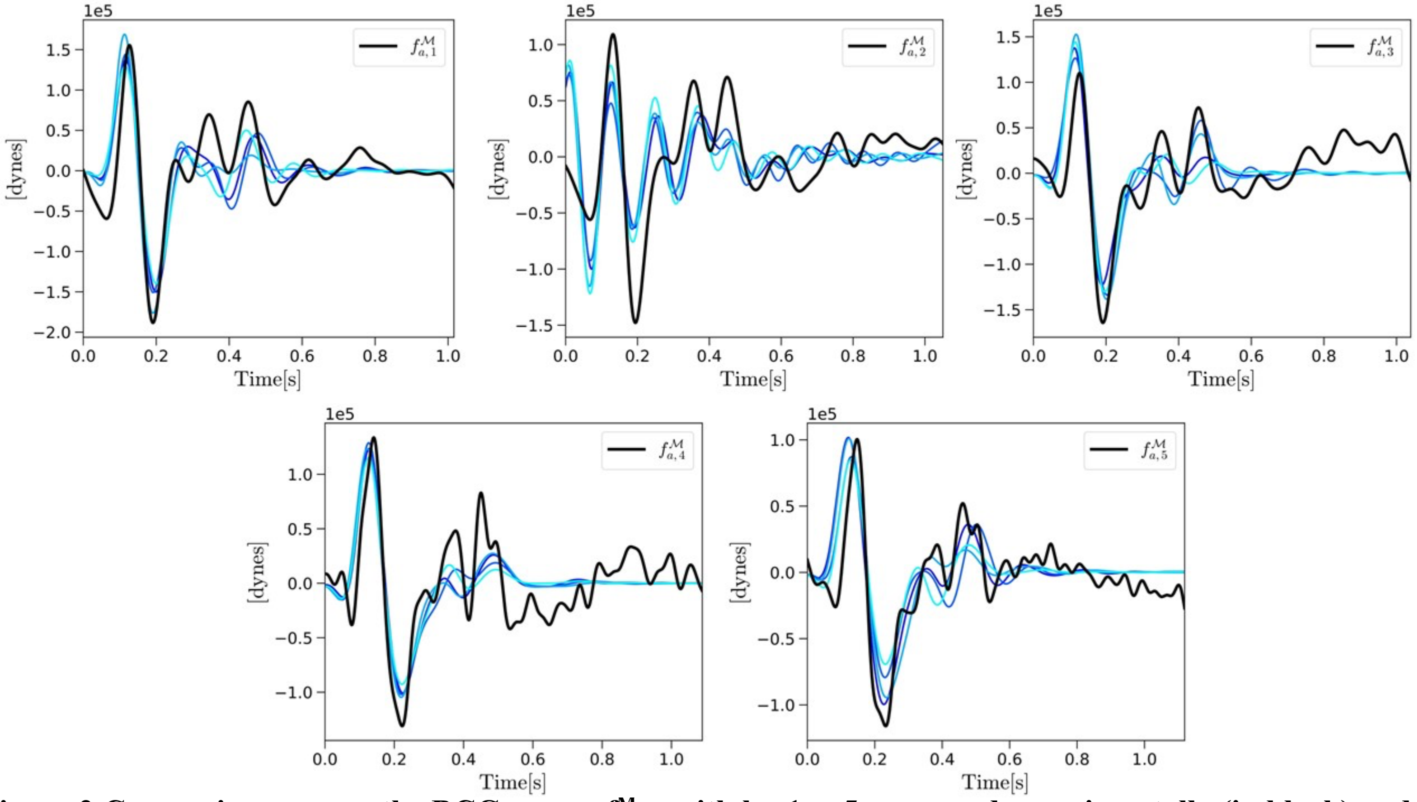

Figure 2 Comparison among the BCG curves $f^{M}{}_{k}$, with k= 1,...,5 measured experimentally (in black) and the corresponding three best-ranked curves computed via the EA (in colors) for Subject 3.

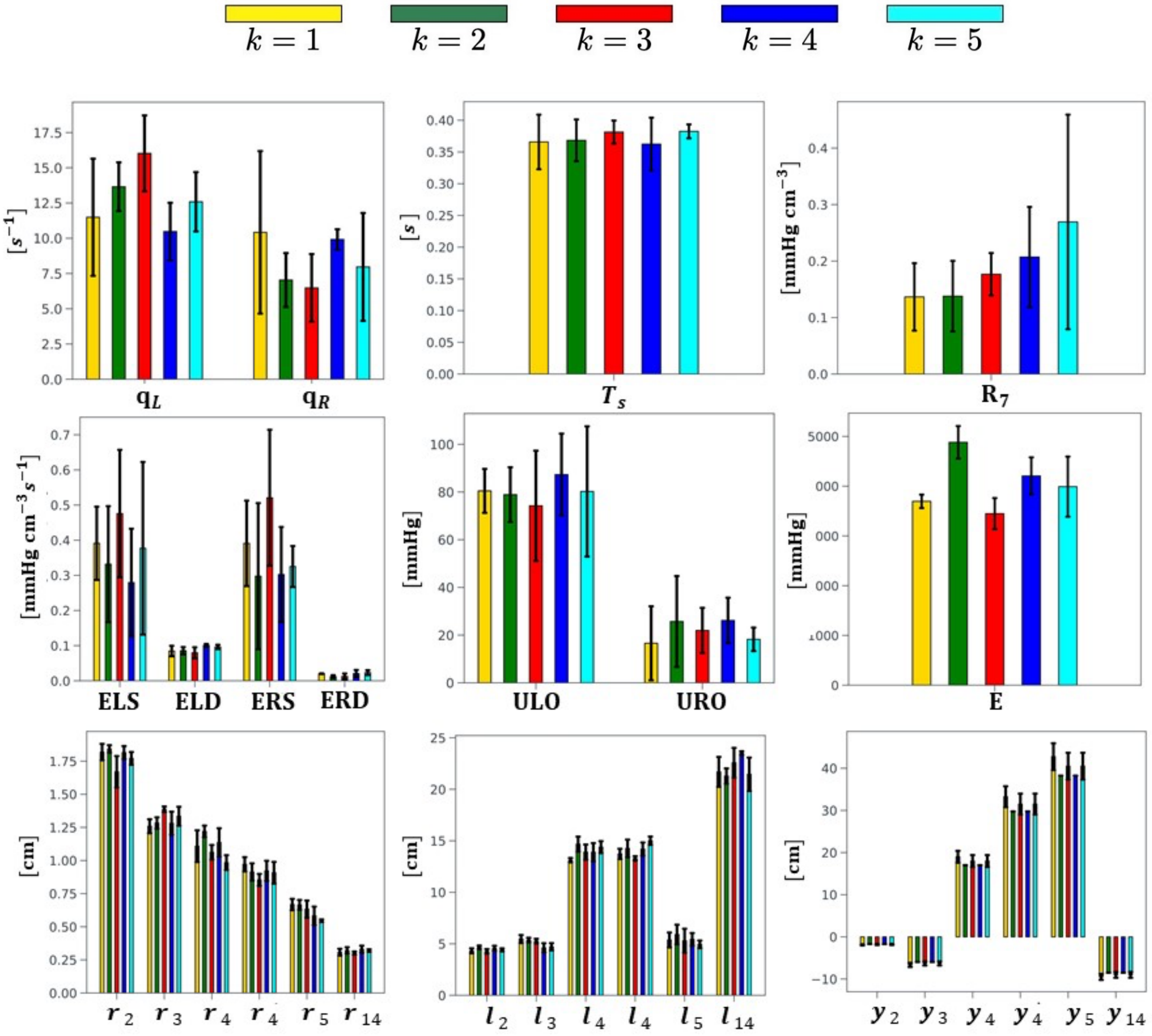

Figure 3 Summary of the physiological and anatomical parameters estimated by the EA for each of the k= 1,...,5 selected BCG objective curves for Subject 2

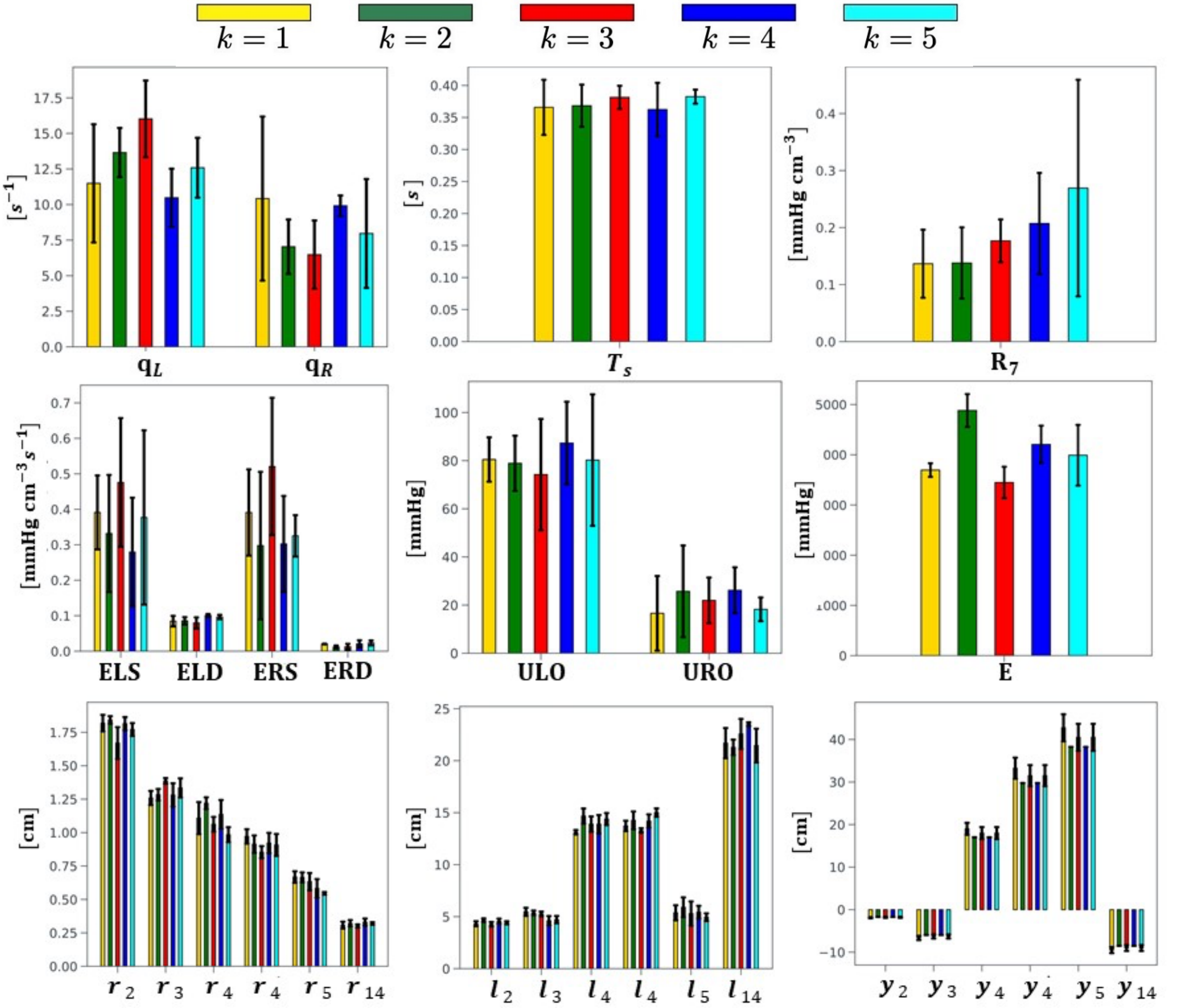

Figure 4 Summary of the physiological and anatomical parameters estimated by the EA for each of the k= 1,...,5 selected BCG objective curves for Subject 3

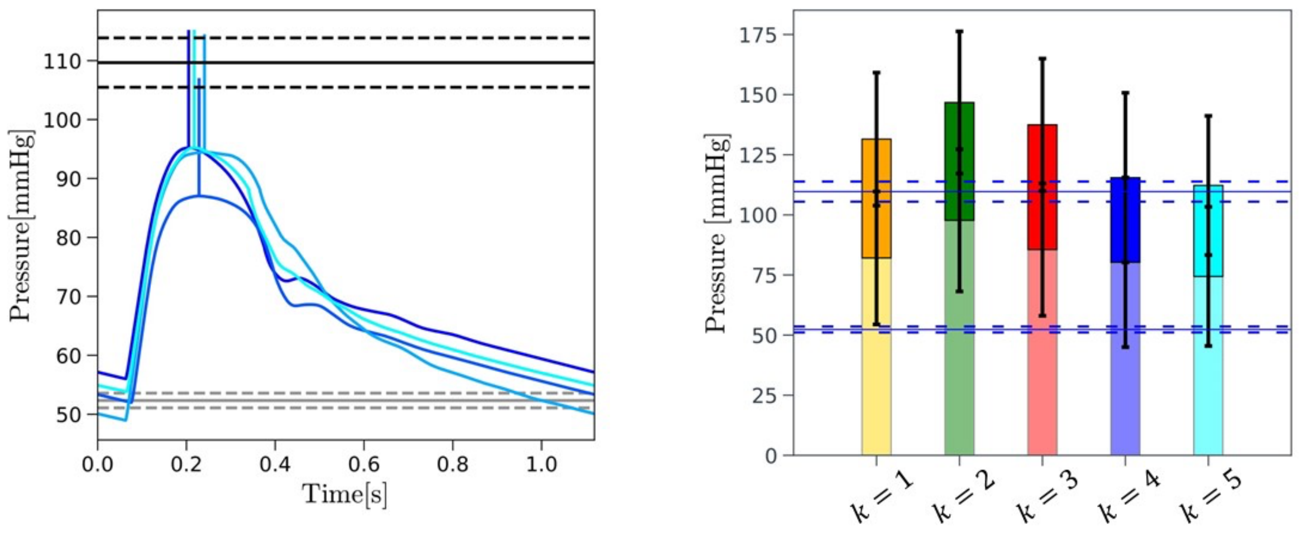

Figure 5 Left: Comparison between the central aortic pressure corresponding to the three best-ranked curves selected by the EA search performed on fM1 for Subject 2 (light blue curves) and the blood pressure measured at the arm with a cuff of the subject (horizontal black lines). The 20 mmHg increment applied to the systolic value of predicted central aortic pressure is also indicated (vertical yellow segments). The mean (solid black lines) and the maximum e minimum values (dashed black lines) of the repeated blood pressure measurements are reported. Right: Comparison between the experimentally measured brachial pulse pressure (horizontal blue lines) and the brachial pressure predicted by the EA for each of the k= 1,...,5 objective curves for Subject 2. The pulse pressure is indicated with solid colors. Maximum and minimum values obtained for the three best-ranked curves for each $f^M{}_k$, with k= 1,...,Nc, are reported in black brackets.

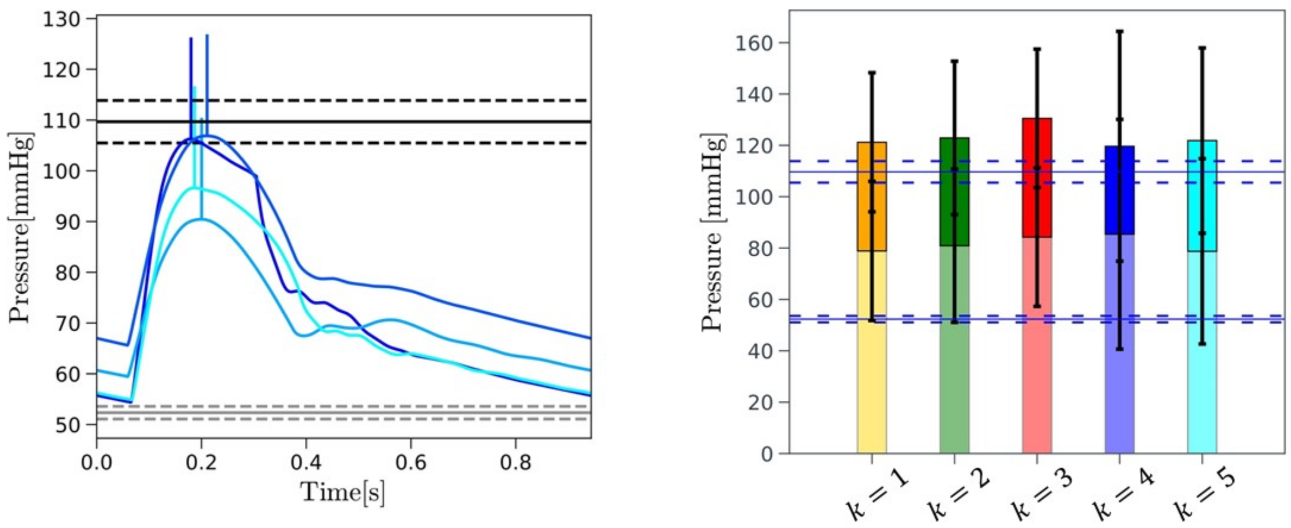

Figure 6 Left: Comparison between the central aortic pressure corresponding to the three best-ranked curves selected by the EA search performed on fM1 for Subject 3 (light blue curves) and the blood pressure measured at the arm with a cuff of the subject (horizontal black lines). The 20 mmHg increment applied to the systolic value of predicted central aortic pressure is also indicated (vertical yellow segments). The mean (solid black lines) and the maximum e minimum values (dashed black lines) of the repeated blood pressure measurements are reported. Right: Comparison between the experimentally measured brachial pulse pressure (horizontal blue lines) and the brachial pressure predicted by the EA for each of the k= 1,...,5 objective curves for Subject 3. The pulse pressure is indicated with solid colors. Maximum and minimum values obtained for the three best-ranked curves for each f^{M}_{k}, with k= 1,...,Nc, are reported in black brackets.
